# New Heparanase-Inhibiting Triazolo-Thiadiazoles Attenuate Primary Tumor Growth and Metastasis

**DOI:** 10.3390/cancers13122959

**Published:** 2021-06-13

**Authors:** Uri Barash, Shobith Rangappa, Chakrabhavi Dhananjaya Mohan, Divakar Vishwanath, Ilanit Boyango, Basappa Basappa, Israel Vlodavsky, Kanchugarakoppal S. Rangappa

**Affiliations:** 1 Technion Integrated Cancer Center (TICC), the Rappaport Faculty of Medicine, Technion, Haifa 31096, Israel; ubarash@yahoo.com (U.B.); bilanit@rambam.health.gov.il (I.B.); 2 Adichunchanagiri Institute for Molecular Medicine, BG Nagara, Nagamangala Taluk 571448, India; shobithrangappa@gmail.com; 3 Department of Studies in Molecular Biology, University of Mysore, Manasagangotri, Mysore 570006, India; cd.mohan@yahoo.com; 4 Laboratory of Chemical Biology, Department of Studies in Organic Chemistry, University of Mysore, Manasagangotri, Mysore 570006, India; divakardivi166@gmail.com (D.V.); salundibasappa@gmail.com (B.B.); 5 Institution of Excellence, Vijnana Bhavan, University of Mysore, Manasagangotri, Mysore 570006, India

**Keywords:** triazolo–thiadiazoles, small molecules, heparanase-inhibiting compounds, primary tumor growth, cell invasion, metastasis

## Abstract

**Simple Summary:**

Heparanase is an endoglycosidase that plays a critical role in tumor progression and metastasis. The expression of heparanase in the tumor microenvironment is positively correlated with the aggressiveness of the tumor and is associated with poor prognosis. In this study, we have demonstrated that a new triazole–thiadiazole-bearing small molecule showed good heparanase inhibition along with attenuation of tumor growth and metastasis. To the best of our knowledge, this is the first report showing a marked decrease in primary tumor growth in mice treated with a small molecule that inhibits heparanase enzymatic activity. Given these encouraging results, studies are underway to better elucidate the mode of action and clinical significance of triazolo–thiadiazoles.

**Abstract:**

Compelling evidence ties heparanase, an endoglycosidase that cleaves heparan sulfate side (HS) chains of proteoglycans, with all steps of tumor development, including tumor initiation, angiogenesis, growth, metastasis, and chemoresistance. Moreover, heparanase levels correlate with shorter postoperative survival of cancer patients, encouraging the development of heparanase inhibitors as anti-cancer drugs. Heparanase-inhibiting heparin/heparan sulfate-mimicking compounds and neutralizing antibodies are highly effective in animal models of cancer progression, yet none of the compounds reached the stage of approval for clinical use. The present study focused on newly synthesized triazolo–thiadiazoles, of which compound 4-iodo-2-(3-(p-tolyl)-[1,2,4]triazolo[3,4-b][1,3,4]thiadiazol-6-yl)phenol (4-MMI) was identified as a potent inhibitor of heparanase enzymatic activity, cell invasion, experimental metastasis, and tumor growth in mouse models. To the best of our knowledge, this is the first report showing a marked decrease in primary tumor growth in mice treated with small molecules that inhibit heparanase enzymatic activity. This result encourages the optimization of 4-MMI for preclinical and clinical studies primarily in cancer but also other indications (i.e., colitis, pancreatitis, diabetic nephropathy, tissue fibrosis) involving heparanase, including viral infection and COVID-19.

## 1. Introduction

The extracellular matrix (ECM) is a supramolecular network of macromolecules (i.e., collagens, glycoproteins proteoglycans) that provide structural and biochemical support to the cellular compartment of tissues [1,2]. Heparan sulfate (HS) is a linear glycosaminoglycan polysaccharide made up of tandem disaccharide units of glucuronic acid or iduronic acid and D-glucosamine, which are covalently attached to a core protein (i.e., syndecan, glypican, perlecan) together forming heparan sulfate proteoglycans (HSPG) [3,4]. Although there are only two repeating sugar units, they exhibit broad diversity in structure generated by a complex pattern of deacetylation, sulfation, and epimerization [3]. Importantly, the sulfated regions of HS serve as docking sites for ECM components, extrinsic proteins, growth-promoting factors (i.e., VEGF, FGF, HGF, TGFβ), chemokines, and cytokines [4,5], indicating that HSPG is crucial in maintaining tissue/cell architecture, growth, and differentiation [3,6]. Heparanase is the only β-endoglycosidase that partially degrades the HS saccharide side chains of HSPG to generate fragments of 4–7 kDa in length [7,8,9]. The degradation products of HS and, even more so, the liberated biologically active molecules, serve as a bioavailable source of HS-bound proteins that activate signal transduction and promote tissue remodeling, repair, neovascularization, and growth [8,9]. Compelling evidence gathered during the last two decades tie heparanase levels not only with tumor metastasis but with all steps of tumor development, including tumor initiation, angiogenesis, growth, metastasis, and chemoresistance [8,9]. Moreover, heparanase levels correlate with shorter postoperative survival of cancer patients [10,11,12,13] altogether indicating that heparanase is causally involved in cancer progression, encouraging the development of heparanase inhibitors as anti-cancer drugs. Heparanase-inhibiting heparin/HS-mimicking compounds, small molecules, and antibodies are being developed [14,15,16], of which several saccharide-based compounds were and/or are being examined in cancer clinical trials [17,18], yet none of the compounds reached the stage of approval for clinical use. The present study is a continuation of our previously reported in vitro screening of a library of small molecules characterized by a variety of scaffolds [19]. It focuses on newly developed triazolo–thiadiazole small molecules selected out of nearly 150 compounds [19], including 10 newly synthesized triazole amino thiol derivatives of substituted [1,2,4]triazolo[3,4-b][1,3,4]thiadiazoles. Our compound of choice {4-iodo-2-(3-(p-tolyl)-[1,2,4]triazolo[3,4-b][1,3,4]thiadiazol-6-yl)phenol = 4-MMI} [19] was found to efficiently inhibit heparanase enzymatic activity, cell invasion, experimental metastasis, and tumor growth in mouse models. To the best of our knowledge, this is the first report showing a marked decrease in primary tumor growth in mice treated with small molecules that inhibit heparanase enzymatic activity.

## 2. Materials and Methods

All chemicals and solvents were purchased from Sigma-Aldrich (Bangalore, India). The completion of the reaction was monitored by pre-coated silica gel TLC plates. An Agilent mass spectrophotometer was used to record the mass of the synthesized compounds. ^1^H and ^13^C NMR were recorded on Agilent and Jeol NMR spectrophotometers (400 MHz). TMS was used as an internal standard, and DMSO was used as a solvent. Chemical shifts are expressed as ppm.

### 2.1. General Procedure for the Synthesis of 4-amino-5-substituted phenyl-4H-1,2,4-triazole-3-thiol (4a–e)

A mixture of ethyl benzoate (1 mmol) in 15 mL of ethanol, as well as hydrazine hydrate (1 mmol), was added and refluxed for 5 h. Completion of the reaction was monitored by TLC followed by a water wash to remove traces of hydrazine hydrate, and acid hydrazide was obtained as a white solid. The acid hydrazide (1 mmol) was added to a stirred solution of ethanol (15 mL) containing KOH (2 mmol), and CS_2_ (1.3 mmol) was added and refluxed for 10 h. After completion of the reaction as monitored by TLC, diethyl ether was added and stirred for 1 h. The potassium salt of hydrazine carbothioate was obtained as a solid, filtered, and washed with diethyl ether. The above potassium salt (1 mmol) was dissolved in 15 mL of water, and hydrazine hydrate (2 mmol) was added and refluxed at 100 °C for 4 h, during which H_2_S evolved and the color of the reaction mass turned to green. Then, the reaction mass was cooled and acidified with concentrated HCl. The solid obtained was recrystallized in ethanol to obtain pure triazole. The reaction progress was monitored using TLC.

### 2.2. General Procedure for the Synthesis of 6-substituted-(3-substituted phenyl-[1,2,4]triazolo[3,4-b][1,3,4]thiadiazole)

To a mixture of 4-amino-5-substituted phenyl-4H-1,2,4-triazole-3-thiol (1 mmol) and substituted salicylic acid (1 mmol), 6 mL of POCl_3_ was added and refluxed at 80 °C for 8 h. The completion of the reaction was monitored by TLC. The reaction mass was quenched with crushed ice and neutralized by potassium carbonate to pH 8. The solid obtained was filtered, washed with water, and recrystallized with an appropriate solvent to obtain 6-substituted-(3-substituted-[1,2,4]triazolo[3,4-b][1,3,4]thiadiazole).

### 2.3. 2,4-diiodo-6-(3-(4-nitrophenyl)-[1,2,4]triazolo[3,4-b][1,3,4]thiadiazol-6-yl)phenol (4-NDI)

Yellow solid. Yield: 70%. ^1^H NMR (DMSO, 400 MHz): δ 9.04 (s, 1H), 8.73 (s, 1H), 8.54–8.36 (m, 2H), 8.19 (s, 1H), 7.96 (s, 1H). ^13^C NMR (DMSO, 100 MHz): δ 164.14, 163.23, 150.27, 148.23, 138.30, 131.76, 131.15, 126.79, 125.24, 124.71, 120.15, 95.80, 90.81. LCMS: 590.8359 (*m*/*z*), 591.7344 [M+1]^+^.

### 2.4. 2-(3-(3-bromophenyl)-[1,2,4]triazolo[3,4-b][1,3,4]thiadiazol-6-yl)-4,6-diiodophenol (3-BDI)

Brown solid. Yield: 81%. ^1^H NMR (DMSO, 400 MHz): δ 8.41(s, 1H), 8.33 (d, *J* = 8 Hz, 1H), 8.09 (d, *J* = 1.6 Hz, 1H), 7.85 (d, *J* = 1.6 Hz, 1H), 7.69 (d, *J* = 7.6 Hz, 1H), 7.64–7.50 (m, 1H). ^13^C NMR (DMSO, 100 MHz): δ 166.49, 165.79, 150.41, 147.60 (2C), 134.22, 132.74, 131.99, 129.09, 128.33, 124.95, 122.76, 116.01, 98.33, 70.09. LCMS: 623.7613 (*m*/*z*), 624.5974 [M+1]^+^.

### 2.5. 2-(3-(3-chlorophenyl)-[1,2,4]triazolo[3,4-b][1,3,4]thiadiazol-6-yl)-4,6-diiodophenol (3-CDI)

Pale yellow solid. Yield: 75%. ^1^H NMR (DMSO, 400 MHz): δ 8.27(s, 2H), 7.76 (s, 1H), 7.68 (d, *J* = 7.6 Hz, 1H), 7.61 (d, *J* = 7.6 Hz, 1H), 7.56 (d, *J* = 7.6 Hz, 1H). ^13^C NMR (DMSO, 100 MHz): δ 168.66, 167.62, 159.54, 151.40, 149.52, 136.42, 134.36, 131.86, 131.75, 127.45, 126.80, 126.35, 116.0, 90.50, 82.69. LCMS: 579.8118 (*m*/*z*), 580.6468 [M+1]^+^.

### 2.6. 2,4-diiodo-6-(3-(4-chlorophenyl)-[1,2,4]triazolo[3,4-b][1,3,4]thiadiazol-6-yl)phenol (4-CDI)

Yellow solid. Yield: 78%. ^1^H NMR (DMSO, 400 MHz): δ 8.30 (d, *J* = 8.4 Hz, 2H), 8.23 (s, 1H), 8.06 (s, 1H), 7.68 (d, *J* = 8 Hz, 2H). ^13^C NMR (DMSO, 100 MHz): δ 163.50, 160.67, 148.17 (2C), 134.38 (2C), 129.24, 127.53, 127.32, 124.78, 117.98, 95.15, 80.83. LCMS: 579.8118 (*m*/*z*), 580.6678 [M+1]^+^.

### 2.7. 2,4-diiodo-6-(3-(p-tolyl)-[1,2,4]triazolo[3,4-b][1,3,4]thiadiazol-6-yl)phenol (4-MDI)

Yellow solid. Yield: 80%. ^1^H NMR (DMSO, 400 MHz): δ 8.18 (d, *J* = 8 Hz, 2H), 8.08 (s, 1H), 7.81 (s, 1H), 7.39 (d, *J* = 7.2 Hz, 2H), 2.36 (s, 3H). ^13^C NMR (DMSO, 100 MHz): δ 166.94, 165.46, 147.37, 139.75, 134.09, 130.20, 128.11, 126.43, 126.11, 124.28, 115.97, 98.61, 69.22, 21.65. LCMS: 559.8665 (*m*/*z*), 558.8661 [M-1]^+^.

### 2.8. 4-iodo-2-(3-(4-nitrophenyl)-[1,2,4]triazolo[3,4-b][1,3,4]thiadiazol-6-yl)phenol (4-NMI)

Off-white solid. Yield: 76%. ^1^H NMR (DMSO, 400 MHz): δ 9.00 (s, 1H), 8.62 (d, *J* = 7.6 Hz, 1H), 8.39–8.25 (m, 2H), 7.96–7.82 (m, 2H), 7.67 (d, *J* = 7.2 Hz, 1H). ^13^C NMR (DMSO, 100 MHz): δ 161.88, 161.08, 156.03, 148.04, 141.86, 134.76, 131.41, 130.97, 126.95, 124.30, 119.85, 119.26, 82.00. LCMS: 464.9393 (*m*/*z*), 465.9448 [M+1]^+^.

### 2.9. 2-(3-(3-bromophenyl)-[1,2,4]triazolo[3,4-b][1,3,4]thiadiazol-6-yl)-4-iodophenol (3-BMI)

Off-white solid. Yield: 84%. ^1^H NMR (DMSO, 400 MHz): δ 8.30 (d, *J* = 8.8 Hz, 2H), 8.20 (d, *J* = 7.6 Hz, 1H), 7.85 (d, *J* = 8 Hz, 1H), 7.70 (d, *J* = 8 Hz, 1H), 7.43 (d, *J* = 8.4 Hz, 1H), 7.61–7.47 (m, 1H). ^13^C NMR (DMSO, 100 MHz): δ 160.85, 156.12, 150.53, 141.74, 135.21, 132.72, 131.24, 127.92, 127.47, 124.49, 122.31, 122.19, 121.12, 119.36, 87.18. LCMS: 497.8647 (*m*/*z*), 498.8651 [M+1]^+^.

### 2.10. 2-(3-(3-chlorophenyl)-[1,2,4]triazolo[3,4-b][1,3,4]thiadiazol-6-yl)-4-iodophenol (3-CMI)

Yellow solid. Yield: 81%. ^1^H NMR (DMSO, 400 MHz): δ 8.38–8.23 (m, 2H), 8.09 (d, *J* = 2.4 Hz, 1H), 7.75–7.59 (m, 1H), 7.58 (d, *J* = 7.2 Hz, 1H), 7.34–7.22 (m, 1H), 6.35 (d, *J* = 8.8 Hz, 1H). ^13^C NMR (DMSO, 100 MHz): δ 168.55, 167.57, 159.48, 151.38, 149.47, 136.31, 134.32, 131.87, 131.75, 130.28, 127.41, 126.69, 126.31, 115.85, 90.63. LCMS: 453.9152 (*m*/*z*), 454.9253 [M+1]^+^.

### 2.11. 2-(3-(4-chlorophenyl)-[1,2,4]triazolo[3,4-b][1,3,4]thiadiazol-6-yl)-4-iodophenol (4-CMI)

Yellow solid. Yield: 80%. ^1^H NMR (DMSO, 400 MHz): δ 8.27 (d, *J* = 8.4 Hz, 1H), 7.98 (d, *J* = 2.4 Hz, 1H), 7.77 (d, *J* = 8.8 Hz, 1H), 7.75–7.63 (m, 2H), 6.83 (d, *J* = 8.8 Hz, 1H), 7.55 (d, *J* = 8.4 Hz, 1H). ^13^C NMR (DMSO, 100 MHz): δ 168.43, 164.73, 157.79, 150.08, 142.76, 138.69, 136.34, 129.66, 128.07, 124.24, 120.28, 81.56. LCMS: 453.9152 (*m*/*z*), 454.9253 [M+1]^+^.

### 2.12. 4-iodo-2-(3-(p-tolyl)-[1,2,4]triazolo[3,4-b][1,3,4]thiadiazol-6-yl)phenol (4-MMI)

Yellow solid. Yield: 84%. ^1^H NMR (DMSO, 400 MHz): δ 8.22 (s, 1H), 8.19–8.00 (m, 2H), 7.71 (d, *J* = 9.2 Hz, 1H), 7.52 (d, *J* = 8.8 Hz, 1H), 7.46–7.29 (m, 2H), 2.35 (s, 3H). ^13^C NMR (DMSO, 100 MHz): δ 161.14, 158.33, 150.56, 148.22, 140.24, 135.20, 130.15, 130.05, 126.26, 126.19, 123.28, 121.42, 84.01, 21.51. LCMS: 433.9698 (*m*/*z*), 434.8964 [M+1]^+^.

### 2.13. Cells and Cell Culture

U87 human glioma cells were purchased from the American Type Culture Collection. (ATCC). MPC-11 mouse myeloma cells were kindly provided by Dr. Ralph Sanderson (the University of Alabama at Birmingham, Birmingham, AL, USA) [20] and luciferase-labeled 4T1 mouse breast carcinoma cells were kindly provided by Dr. Yuval Shaked (TICC, Technion, Haifa, Israel) [21]. U87 and 4T1cells were cultured in Dulbecco’s modified Eagle’s medium (DMEM) and the MPC-11 cells in RPMI medium, supplemented with glutamine, pyruvate, antibiotics, and 10% fetal calf serum (Biological Industries, Beit Haemek, Israel) in a humidified atmosphere containing 5% CO_2_ at 37 °C.

### 2.14. Colorimetric Heparanase Assay

The assay was carried out in a 96-well plate in which the formation of the disaccharide product upon cleavage of fondaparinux (Arixtra, GlaxoSmithKline, Brentford, UK) was estimated using WST-1 tetrazolium salt as described earlier [22,23]. In brief, the reaction system (100 μL) comprised sodium acetate buffer (pH 5.0, 40 mM) and fondaparinux (100 mM) with or without different doses of chemicals under investigation. The assay was initiated by the addition of recombinant heparanase to make the final concentration of 140 pM. The reaction vessels were placed at 37 °C for 18 h, followed by the addition of stop solution (100 μL). WST-1 prepared in 0.1 M NaOH served as a stop solution. Thereafter, the plates were incubated at 60 °C for 60 min, and absorbance was measured at 584 nm. N-(4-([4-(1H-Benzoimidazol-2-yl)-arylamino]-methyl)-phenyl)-benzamide (compound #98) was used as a reference compound. The title compounds were examined for their inhibitory efficacy towards heparanase at 10 and 50 μg/mL. Compounds that yielded inhibition at these concentrations were further tested at lower concentrations to determine their IC_50_.

### 2.15. ECM Degradation Heparanase Assay

ECM degradation heparanase assay was carried out as reported earlier [24,25,26]. Briefly, ECM labeled with sulfate [^35^S] [25] and coating the surface of 35-mm cultures plates was treated with heparanase (200 ng/mL) with or without compounds under investigation. The medium was collected to examine the cleavage of HS proteoglycans and subjected to molecular sieving on Sepharose 6B columns. The radioactivity was measured in the eluted fractions (0.2 mL). Blue dextran and phenol red were used to mark the excluded volume (Vo) and included volume (Vt), respectively. The cleaved HS chains were eluted at 0.5 < Kav < 0.8 (fractions 16–32), and the nearly intact proteoglycan was eluted with the void volume [24]. Each experiment was carried out thrice and variation in elution positions did not exceed ±15%.

### 2.16. Cell Invasion

Invasion assay was performed using Boyden chambers as reported previously [27,28]. Briefly, U87 cells (5 × 10^5^) were seeded into the upper chamber of the invasion chamber in a medium devoid of serum, and the lower chamber contained media supplemented with serum. The cell invasion on treatment with title compounds (4-MMI, 4-CMI; 10 mg/mL) was analyzed by staining with 0.5% crystal violet (Sigma-Aldrich) and photographed.

### 2.17. Experimental Metastasis

4TI mouse breast cancer cells tagged with luciferase (1.5 × 10^5^/0.1 mL/BALB/c mouse) were inoculated intravenously [21,29]. Test compounds (i.p., 500 μg/mouse) or DMSO (0.1 mL/mouse) were administered 20 min before cell injection. The mice were analyzed using IVIS bioluminescent on day 7 and day 13 post-inoculation. These cells metastasize primarily to the lungs.

### 2.18. IVIS Imaging

The bioluminescence imaging of tumors generated from luciferase-expressing cancer cells was carried out using a highly sensitive, cooled charge-coupled device camera assembled in a specimen box (IVIS; Xenogen Corp, Hopkinton, MA, USA). The imaging has advantages such as real-time capture, non-invasive, and quantitative values. For this, the tumor-bearing mice were intraperitoneally administered with a luciferase substrate D-luciferin at a dose of 150 mg/kg body weight. The experimental animal was anesthetized and kept in a camera box followed by continuous exposure to isoflurane (EZAnesthesia, Palmer, PA, USA). The bioluminescence from the tumor mass was detected by the IVIS system, and, subsequently, the tumor burden was quantified using Living Image software (Xenogen) [30,31].

### 2.19. MPC-11 Tumor Growth

The mouse myeloma (MPC-11) cells were gently washed with PBS and the cell number was adjusted to 5 × 10^6^ cells/mL. Thereafter, six-week-old female BALB/c mice (*n* = 6) were subcutaneously injected with washed MPC-11 cells (5 × 10^5^/0.1 mL). The experimental animals were treated with either DMSO alone (0.1 mL/mouse) or 4-MMI (daily 200 μg/mouse, i.p.) starting from day 2 after the implantation of tumor cells, for 12 days. The tumor size was measured using calipers on day 8 and day 12. The tumor volume (V) was determined using the equation V = L × W^2^ × 0.52, where L is the length and W is the width of the tumor. Lastly, the mice were euthanized and xenograft tumors were removed, weighed, and fixed using formalin for further studies [32].

### 2.20. Statistics

Data are presented as means ± SE. Statistical significance was analyzed by the 2-tailed Student’s t-test. Values of *p* < 0.05 were considered significant. Data sets passed D’Agostino–Pearson normality (GraphPad Prism 5 utility software, San Diego, CA, USA). All experiments were repeated at least twice with similar results.

## 3. Results

### 3.1. Synthesis of Title Compounds

**Figure 1:**
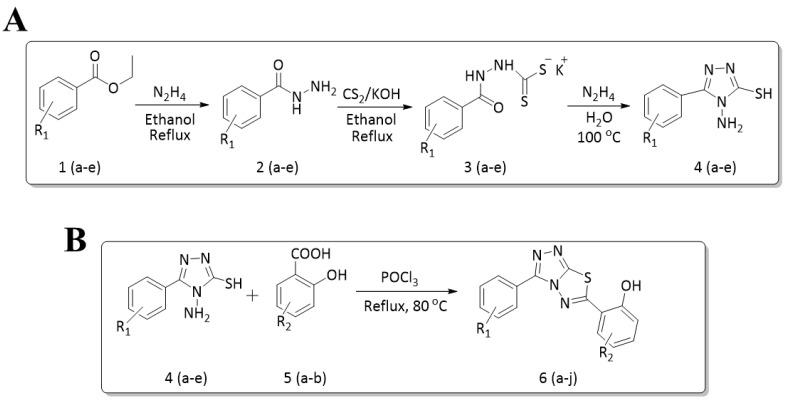
(**A**) Synthesis of 4-amino-5-substituted phenyl-4H-1,2,4-triazole-3-thiol. (**B**) Synthesis of substituted [1,2,4]triazolo[3,4-b] [1,3,4]thiadiazole (where R1 = 4-NO2, 3-Br, 3-Cl, 4-Cl, 4-CH3 and R2 = 3,5-diiodo (5a) and 5-iodo (5b)).

The synthesis of triazolo–thiadiazoles was initiated by the conversion of substituted ethyl benzoates (Figure 1A (1a–e)) to their corresponding hydrazides (2a–e). The esters (1a–e) were refluxed with hydrazine hydrate in ethanol, which resulted in the formation of hydrazides (2a–e) as the intermediates. Compounds 2a–e were treated with carbon disulphide under basic conditions using potassium hydroxide resulted in the formation of potassium salts (3a–e). The potassium salts were further treated with hydrazine to generate 4-amino-5-substituted phenyl-4H-1,2,4-triazole-3-thiols (4a–e). Compounds 4a–e were reacted with substituted salicylic acid in the presence of phosphorous oxychloride under refluxing conditions to obtain triazolo–thiadiazoles, as shown in Figure 1B. The structures of all the target compounds presented in Table 1 were characterized by LCMS, ^1^H NMR, and ^13^C NMR spectrometry.

### 3.2. Newly Synthesized Triazolo–Thiadiazoles Inhibit Heparanase Enzymatic Activity

**Figure 2:**
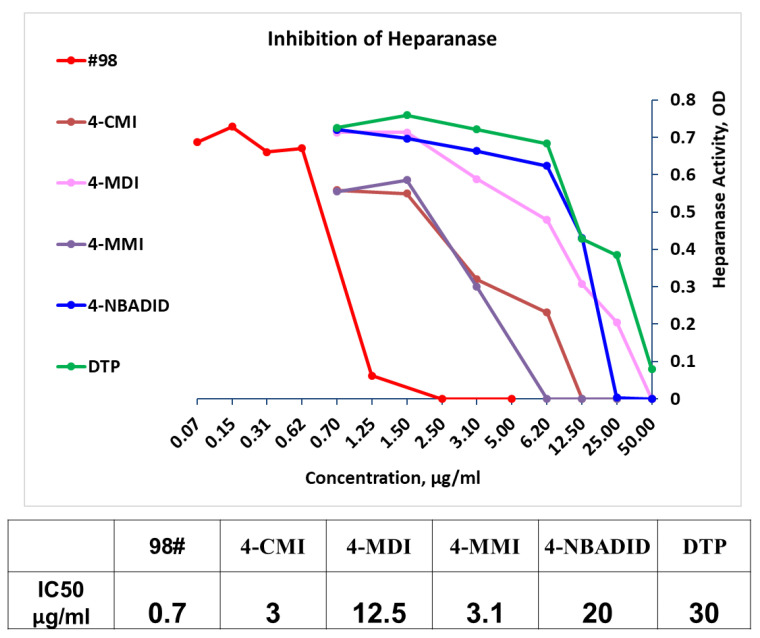
Screening of compounds for inhibition of heparanase enzymatic activity applying the fondaparinux (Arixtra) heparanase assay. Compound #98 (red) = N-(4-phenyl)-benzamide = positive control. Newly synthesized compounds were first tested for inhibition of heparanase at 10 and 50 μg/mL (not shown). Compounds that yielded inhibition at these concentrations were further tested at lower concentrations to determine their IC_50_. Several compounds (i.e., 4-CMI; 4-MMI; 4-MDI; IC_50_ = 3, 3.1, 12.5 μg/mL, respectively) inhibited the enzyme better than 2,4-Diiodo-6-(3-phenyl-[1,2,4]triazolo[3,4-b][1,3,4]thiadiazol-6yl)phenol (DTP; IC_50_ = 30 μg/mL) (bottom table).

The newly synthesized compounds presented in Table 1 were examined for their ability to inhibit the enzymatic activity of recombinant heparanase. For the initial screening of compounds, we applied a colorimetric assay based on the cleavage of the synthetic heparin pentasaccharide fondaparinux (Arixtra) [23]. The assay measures the appearance of a disaccharide product of heparanase-catalyzed fondaparinux cleavage, using the tetrazolium salt WST-1 [23]. All compounds were first tested for inhibition of heparanase at 10 and 50 μg/mL (not shown). Compounds that yielded inhibition at these high concentrations were further examined at lower concentrations to determine their IC_50_ (Figure 2). Among these compounds, 2-(3-(4-chlorophenyl)-[1,2,4]triazolo[3,4-b][1,3,4]thiadiazol-6-yl)-4-iodophenol (4-CMI), 2,4-diiodo-6-(3-(p-tolyl)-[1,2,4]triazolo[3,4-b][1,3,4]thiadiazol-6-yl)phenol (4-MDI), and 4-iodo-2-(3-(p-tolyl)-[1,2,4]triazolo[3,4-b][1,3,4]thiadiazol-6-yl)phenol (4-MMI) showed good heparanase inhibitory activity with IC_50_ values of 3, 12.5, and 3.1 µg/mL, respectively. Compound #98, used as a positive control, displayed an IC_50_ value of 0.7 µg/mL (Figure 2, bottom table).

**Figure 3:**
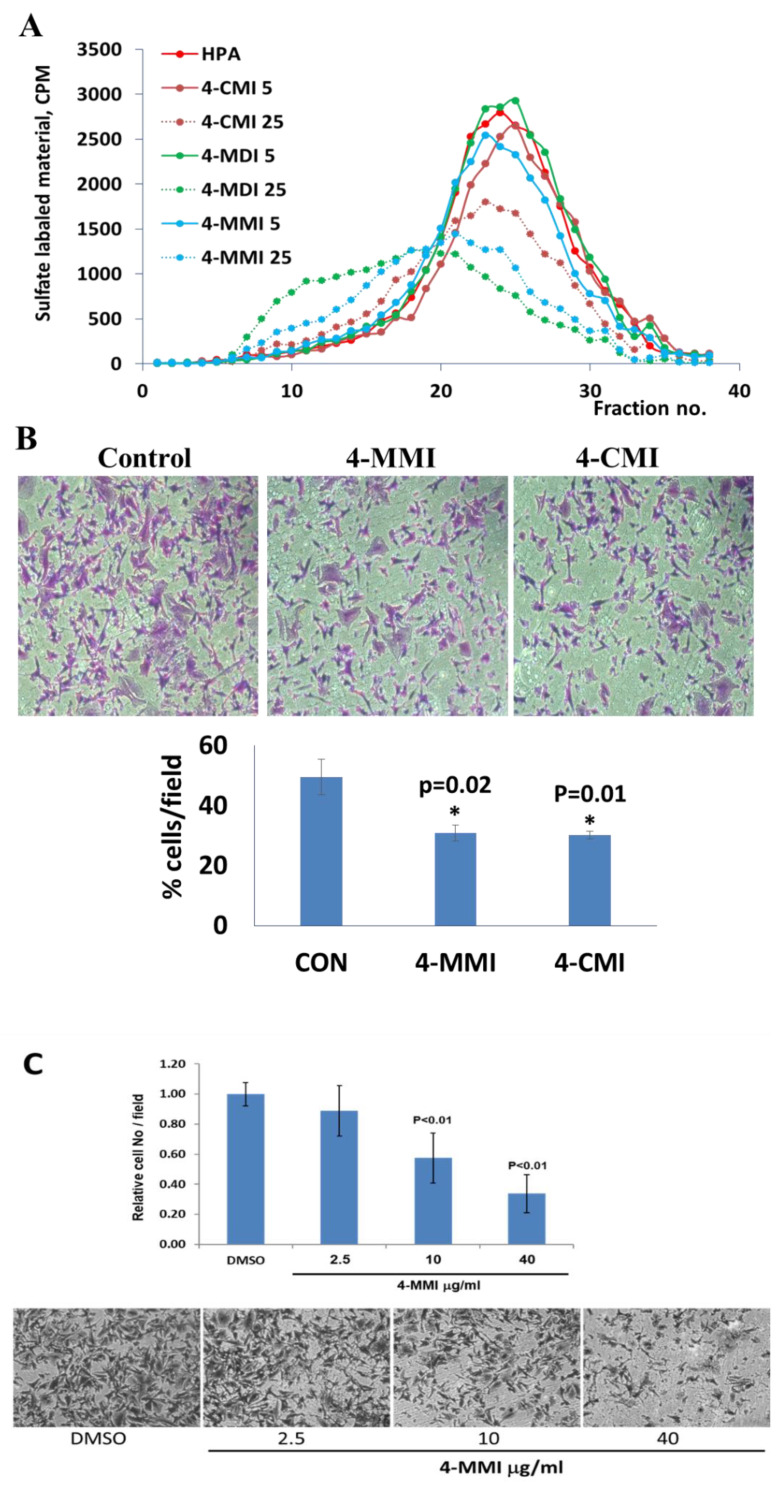
Inhibition of ECM degradation and cell invasion. (**A**) ECM degradation. Active heparanase (200 ng) was incubated for 5 h without (red) or with 5 and 25 µg/mL of 4-CMI, 4-MDI, or 4-MMI on dishes coated with sulfate-labeled ECM. Labeled material released from the ECM was subjected to gel filtration (Sepharose 6B). Heparan sulfate degradation fragments are typically eluted in fractions 18–30. (**B**) Cell invasion. U87 human glioma cells (2 × 10^5^) were plated onto Matrigel-coated 8 μm transwell filters and incubated (6 h, 37 °C) in the absence (Con) or presence of 4-MMI or 4-CMI (10 µg/mL). (**C**) Dose–response showing the effect of compound 4-MMI (2.5, 10, and 40 µg/mL) on U87 cell invasion. Invading cells adhering to the lower side of the membrane were visualized (top panels) and quantified (bottom panel) in 7 random fields as described in ‘Materials and Methods’. The structures of compounds 4-CMI and 4-MMI are presented in Table 1.

To validate the results and better resemble the in vivo situation, we applied metabolically sulfate [Na_2_^35^SO_4_] labeled extracellular matrix (ECM) deposited by cultured endothelial cells. This naturally produced substrate closely resembles the subendothelial basement membrane in its composition, biological function, and barrier properties [25]. Years of experience indicate that compounds that effectively inhibit the enzyme in this assay are also effective in preclinical animal models [9]. This semi-quantitative assay measures release of radioactive heparan sulfate (HS) degradation fragments from an insoluble extracellular matrix (ECM) that is firmly bound to a culture dish [26]. As demonstrated in Figure 3A, compounds 4-CMI, 4-MMI, and 4-MDI inhibited (50–70%) heparanase activity at 25 µg/mL, but there was little or no inhibition at 5 µg/mL.

### 3.3. Compounds 4-CMI and 4-MMI Inhibit Glioma Cell Invasion

The causal involvement of heparanase in cancer metastasis has long been demonstrated in various types of cancer [12,13,24]. Applying Matrigel-coated transwell filters, we investigated the effect of 4-CMI and 4-MMI on human U87 glioma cell invasion. As demonstrated in Figure 3B, treatment with 4-CMI or 4-MMI (10 µg/mL) significantly inhibited the invasion capacity of U87 cells. A similar result was obtained with compound 4-MDI (not shown). A dose–response effect of compound 4-MMI is presented in Figure 3C.

### 3.4. Compounds 4-CMI and 4-MMI Mitigate Breast Carcinoma Experimental Metastasis

**Figure 4:**
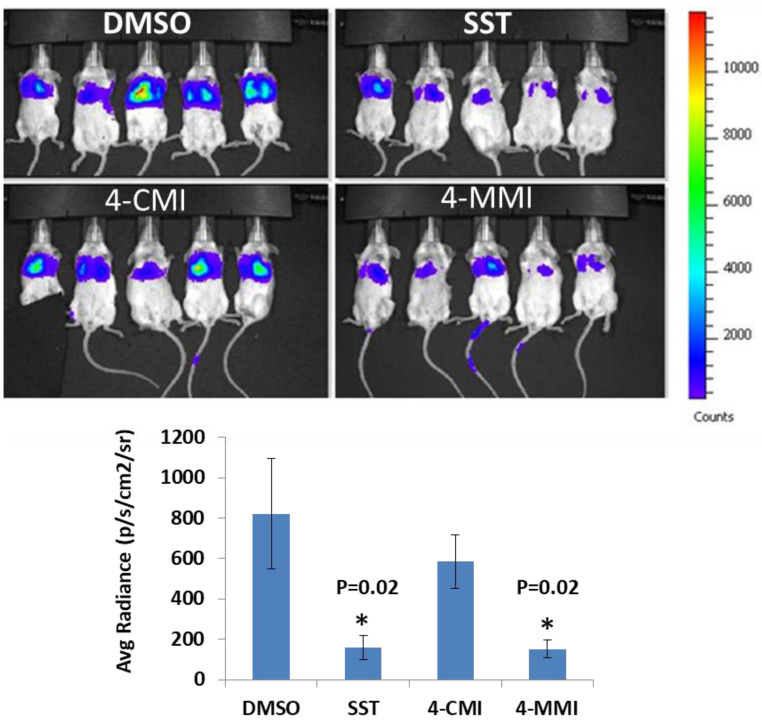
Inhibition of 4T1 breast carcinoma experimental metastasis. Luciferase-labeled 4T1 breast carcinoma cells (1.5 × 10^5^/mouse) were injected i.v. into BALB/c mice (*n* = 6 BALB/c mice/group). Vehicle (DMSO alone), positive control (150 µg/mouse, SST), 4-CMI, or 4-MMI (500 μg/mouse) were injected (i.p.; 0.1 mL/mouse) 20 min prior to cell inoculation. Metastasis was inspected by IVIS on days 7 (not shown) and 13 after cell inoculation. Quantification of the luciferase intensities is shown graphically in the lower panel, as described in ‘Section 2. Materials and Methods’. * *p* = 0.02 (SST vs. DMSO alone); * *p* = 0.02 (4-MMI vs. DMSO alone).

We evaluated the effect of 4-CMI and 4-MMI on 4T1 mouse breast carcinoma experimental metastasis. Briefly, luciferase-labeled 4T1 breast carcinoma cells were injected i.v. into BALB/c mice. Compound 4-CMI or 4-MMI was injected (i.p.) 20 min before cell inoculation, and metastases formation was inspected and quantified by IVIS fluorescence imaging. As demonstrated in Figure 4 and Figure S1, compound 4-MMI and, to a lesser extent, compound 4-CMI inhibited the extravasation of 4T1 cells and their subsequent colonization in the mouse lungs. The effect of 4-MMI was comparable to that exerted by Roneparstat (=SST), a well-characterized N-acetylated, glycol-split heparin [17] that recently completed phase I clinical trial in myeloma patients [33]. These results indicate that triazolo–thiadiazoles attenuate the ability of blood-borne carcinoma cells to extravasate through the subendothelial basement membrane, attributed to their ability to inhibit heparanase enzymatic activity, ECM degradation, and cell invasion.

### 3.5. Compound 4-MMI Reduces Tumor Burden in a Myeloma Model

**Figure 5:**
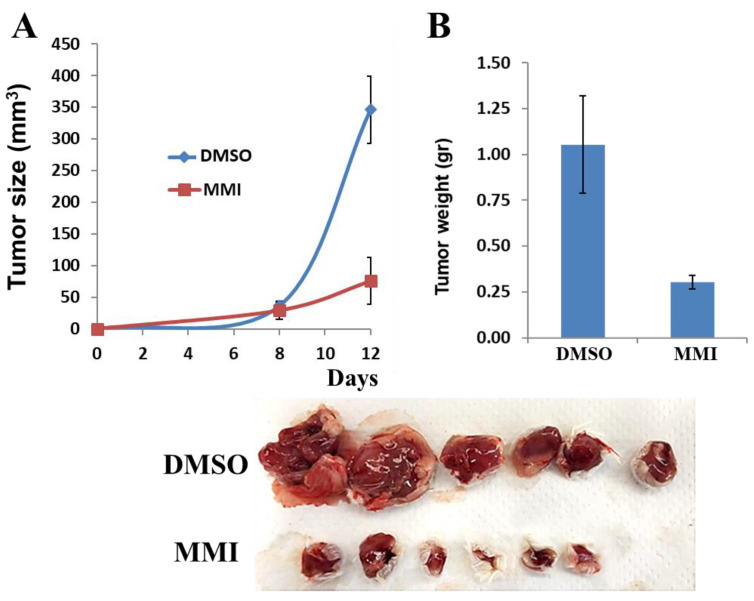
MPC-11 tumor growth. Cells (MPC-11 mouse myeloma) from exponential cultures were washed with PBS and brought to a concentration of 5 × 10^6^ cells/mL. Cell suspension (5 × 10^5^/0.1 mL) was inoculated subcutaneously at the right flank of 6-week-old female BALB/c mice (*n* = 6). Mice were treated with either DMSO (0.1 mL/mouse) or test compound (daily 200 µg/mouse, i.p.), starting on day 2 after the injection of tumor cells, for 12 days. Tumor size was determined on days 8 and 12 by externally measuring the tumors in two dimensions using a caliper (**A**, *p* < 0.001). At the end of the experiment, mice were sacrificed and the tumors resected, weighed (**B**, *p* = 0.003), and photographed.

Next, we examined the capacity of compound 4-MMI to inhibit myeloma primary tumor growth. For this purpose, we applied the MPC-11 syngeneic myeloma model [20,32]. Briefly, BALB/c mice were implanted subcutaneously with MPC-11 mouse myeloma cells (0.5 × 10^6^/mouse) and treated with either DMSO (0.1 mL/mouse) or 4-MMI (daily 200 μg/mouse, i.p.), starting on day two after tumor cell inoculation. Xenograft Tumor size was measured on days 8 and 12 (Figure 5A), resected, and weighed (Figure 5B) (see also Figure S2). Strikingly, compound 4-MMI effectively and markedly decreased the rate of tumor growth and the tumor burden. Immunostaining of fixed paraffin-embedded tissue sections with anti-VEGF antibodies revealed a marked decrease in the levels of VEGF in tumors derived from mice treated with 4-MMI (Figure S3), likely reflecting an anti-angiogenesis effect of the drug.

## 4. Discussion

Heparanase is a multifaceted enzyme that together with heparan sulfate drives signal transduction, immune cell activation, exosome formation, autophagy, and gene transcription via enzymatic and non-enzymatic activities [8,34,35]. Heparanase releases a myriad of HS-bound growth factors, cytokines, and chemokines that are sequestered by heparan sulfate in the glycocalyx and ECM. Collectively, the heparan sulfate–heparanase axis plays pivotal roles in creating a permissive environment for cell proliferation, differentiation, and function, often resulting in the pathogenesis of diseases, such as cancer, chronic inflammation, sepsis, endotheliitis, kidney dysfunction, diabetic nephropathy, diabetes, tissue fibrosis, bone osteolysis, thrombosis, atherosclerosis, and viral infection [36]. Heparanase represents a druggable target because (i) there is only a single enzymatically active heparanase expressed in humans; (ii) the enzyme is present in low levels in normal tissues but is markedly elevated in cancer, inflammation, and other pathologies; and (iii) heparanase deficient mice appear normal [37]. Thus, properly designed heparanase inhibitors will likely have few, if any, negative side effects. The development of heparanase inhibitors has focused predominantly on carbohydrate-based compounds with heparin-like properties [38,39,40]. These compounds bind to the heparin-binding domains that flank the active site of heparanase thereby inhibiting cleavage of heparan sulfate. Four different heparin mimics are currently in clinical trials in human cancer patients. However, all of these mimics have the disadvantage that they are not specific for heparanase and likely interact with different heparin-binding proteins with unknown consequences and off-target effects [38,39]. Therefore, even if they prove efficacious in patients, it will be impossible to attribute their effect solely to heparanase inhibition. Moreover, three of the four mimics are heterogeneous in structure, adding to their complexity and uncertainty as viable drugs for use in humans. Small-molecule inhibitors of heparanase have the potential to overcome some of the limitations of polysaccharides, offering an opportunity for specificity, favorable pharmacokinetics, and oral availability [36,41]. Numerous heparanase-inhibiting small molecules were reported [39,42,43], but none entered clinical testing. Surprisingly, only one compound was found to inhibit experimental metastasis (B16 melanoma model) [44], and there are no reports of their preclinical testing in xenograft tumor models.

We have previously reported the results of an in vitro screening of a library of small molecules characterized by a variety of scaffolds. More than 150 compounds were tested for their ability to inhibit the enzymatic activity of human heparanase, identifying [1,2,4]triazolo[3,4-b][1,3,4]thiadiazole derivatives as active substances. The most potent anti-heparanase derivative, DTP, was found to inhibit tumor cell proliferation, migration, and invasion in vitro with IC_50_ values in the micromolar range [22]. The present study focuses on the development of related triazolo–thiadiazole compounds that inhibit heparanase enzymatic activity, ECM degradation, cell invasion, experimental metastasis, and tumor growth in mouse models. We used DTP as a template structure for the synthesis of substituted triazolo–thiadiazoles endowed with better heparanase inhibitory activity. The structure of DTP was modified by removing iodine at the second position of the phenyl ring and the addition of a methyl group on the terminal benzene ring, which resulted in the formation of compound 4-MMI. MMI showed relatively potent heparanase inhibitory and antitumor activity compared to its parent compound. Other structural analogs showed various degrees of heparanase inhibition. The newly designed compounds were examined for their capacity to inhibit heparanase enzymatic activity, using as substrates heparin-derived pentasaccharide [23] and HS naturally embedded in the ECM produced by endothelial cells [25]. To translate the in vitro inhibition of heparanase enzymatic activity and cell invasion to preclinical models, the most promising triazolo–thiadiazole compounds were examined in the 4T1 mouse mammary carcinoma model of experimental metastasis and the MPC-11 mouse model of myeloma tumor growth [20,32]. Breast cancer metastasis is the leading cause of female mortality worldwide and accounts for 25% of the total number of cancer cases and 15% of all cancer-associated female mortality [45]. Importantly, compound 4-MMI yielded a nearly fourfold inhibition of 4T1 breast carcinoma metastasis, comparable to the effect exerted by Roneparstat (=SST), a well-characterized heparin-like heparanase inhibitor (Figure 4) [15,17,33,36,39,41].

Multiple myeloma is a devastating cancer of plasma cells that is highly dependent on the bone marrow microenvironment for growth and survival [46]. Heparanase has been shown to critically accelerate myeloma tumor growth and bone colonization [47], and the heparanase inhibitor Roneparstat has been examined in myeloma patients, yielding promising results [33]. Strikingly, treatment with compound 4-MMI resulted in a profound inhibition of tumor growth and burden in the aggressive MPC-11 myeloma model (Figure 5). We also noted a profound reduction in VEGF levels in tumor sections derived from 4-MMI treated vs. untreated mice, likely due to inhibition of heparanase-mediated VEGF gene expression and/or release [6,7,10]. Due to limited amounts of the 4-MMI compound, we could not perform dose–response and pharmacokinetic studies. Yet, there were no signs of distress and toxicity in response to the daily administration of 200 ug/mouse/day, a dose that is well below the amounts of anti-cancer small molecules given to mice and the equivalent dose in humans. In subsequent studies we will examine the effect of 4-MMI in additional tumor models, with emphasis on orthotopic models of breast and pancreatic carcinomas. We will also apply medicinal chemistry and computational principles to further optimize the compound for improved potency; specificity; selectivity; and pharmacokinetic properties, including stability, permeability, solubility, and oral bioavailability.

## 5. Conclusions

To the best of our knowledge, this is the first report showing a marked decrease in primary tumor growth in mice treated with a small molecule that inhibits heparanase enzymatic activity. Given these encouraging results, studies are underway to better elucidate the mode of action and clinical significance of compound 4-MMI and related triazolo–thiadiazoles. Among other aspects, the compound is being examined for an effect on human tumor xenografts, primarily myeloma and breast carcinoma, pharmacokinetic characteristics, efficacy, and mode of administration. Docking and SAR studies are being performed, applying the recently resolved crystal structure of the heparanase protein [48,49,50]. Selected molecules exerting little or no side effects will then be examined for oral availability and anti-cancer effects in combination with currently available treatments. We will also study the effect of our lead compounds on other pathological indications involving heparanase, such as chronic inflammation (i.e., colitis, pancreatitis) [51,52], tissue fibrosis [53], kidney dysfunction (i.e., AKI, diabetic nephropathy) [54,55,56,57], and diabetes [58]. Of particular relevance are recent studies on the presumed involvement of heparanase and heparan sulfate in the pathogenesis of COVID-19 [59,60,61,62]. Studies on the effect of 4-MMI and related compounds on SARS-CoV-2 infectivity are ongoing.

## Figures and Tables

**Table 1:** List of synthesized substituted [1,2,4]triazolo[3,4-b] [1,3,4]thiadiazoles.

**Triazole Amino Thiol** **Derivatives**	**Acid**	**Products**
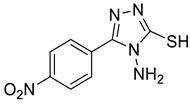 **4a**	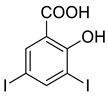 **5a**	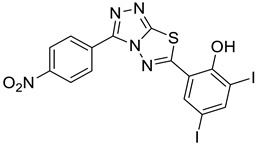 **4-NDI**
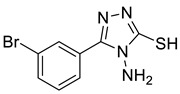 **4b**	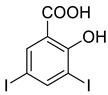 **5a**	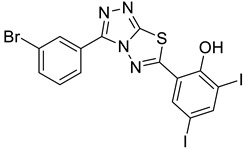 **3-BDI**
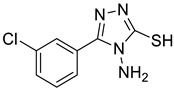 **4c**	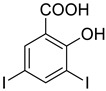 **5a**	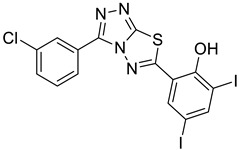 **3-CDI**
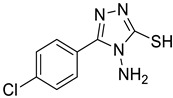 **4d**	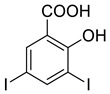 **5a**	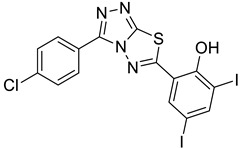 **4-CDI**
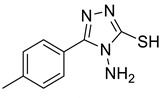 **4e**	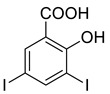 **5a**	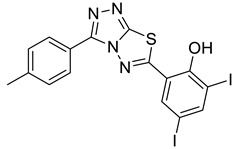 **4-MDI**
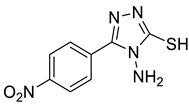 **4a**	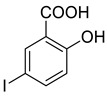 **5b**	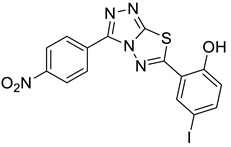 **4-NMI**
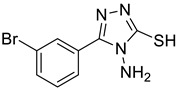 **4b**	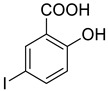 **5b**	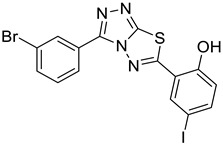 **3-BMI**
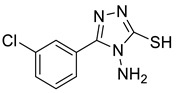 **4c**	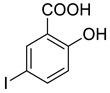 **5b**	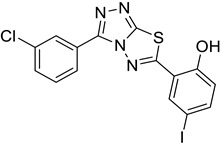 **3-CMI**
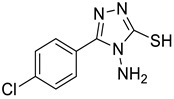 **4d**	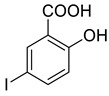 **5b**	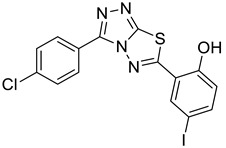 **4-CMI**
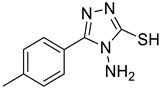 **4e**	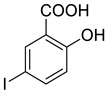 **5b**	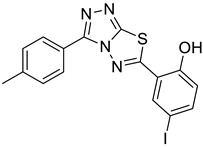 **4-MMI**

## Data Availability

All the data associated with this study are freely available.

## Supplementary Materials

The following are available online at https://www.mdpi.com/article/10.3390/cancers13122959/s1. Figure S1: Inhibition of 4T1 breast carcinoma experimental metastasis, Figure S2: MPC-11 tumor growth, Figure S3: Immunostaining The spectra and other information are freely available with this paper.

## References

[1] Bonnans C., Chou J., Werb Z. (2014). Remodelling the extracellular matrix in development and disease. Nat. Rev. Mol. Cell Biol..

[2] Pozzi A., Yurchenco P.D., Iozzo R.V. (2017). The nature and biology of basement membranes. Matrix Biol..

[3] Karamanos N.K., Piperigkou Z., Theocharis A.D., Watanabe H., Franchi M., Baud S., Brézillon S., Götte M., Passi A., Vigetti D. (2018). Proteoglycan Chemical Diversity Drives Multifunctional Cell Regulation and Therapeutics. Chem. Rev..

[4] Kjellén L., Lindahl U. (2018). Specificity of glycosaminoglycan-protein interactions. Curr. Opin. Struct. Biol..

[5] Basappa, Kumar C.A., Swamy S.N., Sugahara K., Rangappa K.S. (2009). Anti-tumor and anti-angiogenic activity of novel hydantoin derivatives: Inhibition of VEGF secretion in liver metastatic osteosarcoma cells. Bioorganic Med. Chem..

[6] Basappa, Murugan S., Kavitha C.V., Purushothaman A., Nevin K.G., Sugahara K., Rangappa K.S. (2010). A small oxazine compound as an anti-tumor agent: A novel pyranoside mimetic that binds to VEGF, HB-EGF, and TNF-α. Cancer Lett..

[7] Hari S., Swaroop T.R., Preetham H.D., Mohan C.D., Muddegowda U., Basappa S., Vlodavsky I., Sethi G., Rangappa K.S. (2020). Synthesis, Cytotoxic and Heparanase Inhibition Studies of 5-oxo-1-arylpyrrolidine-3- carboxamides of Hydrazides and 4-amino-5-aryl-4H-1,2,4-triazole-3-thiol. Curr. Org. Synth..

[8] Ilan N., Bhattacharya U., Barash U., Boyango I., Yanku Y., Gross-Cohen M., Vlodavsky I. (2020). Heparanase—The Message Comes in Different Flavors. Adv. Exp. Med. Biol..

[9] Vlodavsky I., Ilan N., Sanderson R.D. (2020). Forty Years of Basic and Translational Heparanase Research. Adv. Exp. Med. Biol..

[10] Hao N.-B., Tang B., Wang G.-Z., Xie R., Hu C.-J., Wang S.-M., Wu Y.-Y., Liu E., Xie X., Yang S.-M. (2015). Hepatocyte growth factor (HGF) upregulates heparanase expression via the PI3K/Akt/NF-κB signaling pathway for gastric cancer metastasis. Cancer Lett..

[11] Kundu S., Xiong A., Spyrou A., Wicher G., Marinescu V.D., Edqvist P.-H.D., Zhang L., Essand M., Dimberg A., Smits A. (2016). Heparanase Promotes Glioma Progression and Is Inversely Correlated with Patient Survival. Mol. Cancer Res..

[12] Ilan N., Elkin M., Vlodavsky I. (2006). Regulation, function and clinical significance of heparanase in cancer metastasis and angiogenesis. Int. J. Biochem. Cell Biol..

[13] Vlodavsky I., Beckhove P., Lerner I., Pisano C., Meirovitz A., Ilan N., Elkin M. (2011). Significance of Heparanase in Cancer and Inflammation. Cancer Microenviron..

[14] Mohan C.D., Hari S., Preetham H.D., Rangappa S., Barash U., Ilan N., Nayak S.C., Gupta V.K., Basappa, Vlodavsky I. (2019). Targeting Heparanase in Cancer: Inhibition by Synthetic, Chemically Modified, and Natural Compounds. iScience.

[15] Cassinelli G., Torri G., Naggi A. (2020). Non-Anticoagulant Heparins as Heparanase Inhibitors. Adv. Exp. Med. Biol..

[16] Chhabra M., Ferro V. (2020). PI-88 and Related Heparan Sulfate Mimetics. Adv. Exp. Med. Biol..

[17] Noseda A., Barbieri P. (2020). Roneparstat: Development, Preclinical and Clinical Studies. Adv. Exp. Med. Biol..

[18] Hammond E., Dredge K. (2020). Heparanase Inhibition by Pixatimod (PG545): Basic Aspects and Future Perspectives. Adv. Exp. Med. Biol..

[19] Priya B., Kumar C.A., Swamy S.N., Basappa, Naveen S., Prasad J.S., Rangappa K. (2007). 2-(2-(2-Ethoxybenzoylamino)-4-chlorophenoxy)-*N*-(2-ethoxybenzoyl)benzamine inhibits EAT cell induced angiogenesis by down regulation of VEGF secretion. Bioorganic Med. Chem. Lett..

[20] Sanderson R.D., Sneed T.B., Young L.A., Sullivan G.L., Lander A.D. (1992). Adhesion of B lymphoid (MPC-11) cells to type I collagen is mediated by integral membrane proteoglycan, syndecan. J. Immunol..

[21] Alishekevitz D., Bril R., Loven D., Miller V., Voloshin T., Gingis-Velistki S., Fremder E., Scherer S.J., Shaked Y. (2013). Differential Therapeutic Effects of Anti-VEGF-A Antibody in Different Tumor Models: Implications for Choosing Appropriate Tumor Models for Drug Testing. Mol. Cancer Ther..

[22] Baburajeev C.P., Mohan C.D., Rangappa S., Mason D.J., Fuchs J.E., Bender A., Barash U., Vlodavsky I., Basappa, Rangappa K.S. (2017). S. Identification of Novel Class of Triazolo-Thiadiazoles as Potent Inhibitors of Human Heparanase and their Anticancer Activity. BMC Cancer.

[23] Hammond E., Li C.P., Ferro V. (2010). Development of a colorimetric assay for heparanase activity suitable for kinetic analysis and inhibitor screening. Anal. Biochem..

[24] Vlodavsky I., Friedmann Y., Elkin M., Aingorn H., Atzmon R., Ishai-Michaeli R., Bitan M., Pappo O., Peretz T., Michal I. (1999). Mammalian heparanase: Gene cloning, expression and function in tumor progression and metastasis. Nat. Med..

[25] Vlodavsky I. (1999). Preparation of Extracellular Matrices Produced by Cultured Corneal Endothelial and PF-HR9 Endodermal Cells. Curr. Protoc. Cell Biol..

[26] Vlodavsky I., Fuks Z., Bar-Ner M., Ariav Y., Schirrmacher V. (1983). Lymphoma cell-mediated degradation of sulfated proteoglycans in the subendothelial extracellular matrix: Relationship to tumor cell metastasis. Cancer Res..

[27] Zetser A., Bashenko Y., Miao H.-Q., Vlodavsky I., Ilan N. (2003). Heparanase affects adhesive and tumorigenic potential of human glioma cells. Cancer Res..

[28] Barash U., Spyrou A., Liu P., Vlodavsky E., Zhu C., Luo J., Su D., Ilan N., Forsberg-Nilsson K., Vlodavsky I. (2019). Heparanase promotes glioma progression via enhancing CD24 expression. Int. J. Cancer.

[29] Loka R.S., Sletten E.T., Barash U., Vlodavsky I., Nguyen H.M. (2019). Specific Inhibition of Heparanase by a Glycopolymer with Well-Defined Sulfation Pattern Prevents Breast Cancer Metastasis in Mice. ACS Appl. Mater. Interfaces.

[30] Barash U., Lapidot M., Zohar Y., Loomis C., Moreira A., Feld S., Goparaju C., Yang H., Hammond E., Zhang G. (2018). Involvement of Heparanase in the Pathogenesis of Mesothelioma: Basic Aspects and Clinical Applications. J. Natl. Cancer Inst..

[31] Weissmann M., Arvatz G., Horowitz N., Feld S., Naroditsky I., Zhang Y., Ng M., Hammond E., Nevo E., Vlodavsky I. (2016). Heparanase-neutralizing antibodies attenuate lymphoma tumor growth and metastasis. Proc. Natl. Acad. Sci. USA.

[32] Barash U., Zohar Y., Wildbaum G., Beider K., Nagler A., Karin N., Ilan N., Vlodavsky I. (2014). Heparanase enhances myeloma progression via CXCL10 downregulation. Leukemia.

[33] Galli M., Chatterjee M., Grasso M., Specchia G., Magen H., Einsele H., Celeghini I., Barbieri P., Paoletti D., Pace S. (2018). Phase I study of the heparanase inhibitor roneparstat: An innovative approach for ultiple myeloma therapy. Haematologica.

[34] Vlodavsky I., Singh P., Boyango I., Gutter-Kapon L., Elkin M., Sanderson R.D., Ilan N. (2016). Heparanase: From basic research to therapeutic applications in cancer and inflammation. Drug Resist. Updates.

[35] Vlodavsky I., Gross-Cohen M., Weissmann M., Ilan N., Sanderson R.D. (2018). Opposing Functions of Heparanase-1 and Heparanase-2 in Cancer Progression. Trends Biochem. Sci..

[36] Giannini G., Battistuzzi G., Rivara S. (2020). The Control of Heparanase through the Use of Small Molecules. Adv. Exp. Med. Biol..

[37] Zcharia E., Jia J., Zhang X., Baraz L., Lindahl U., Peretz T., Vlodavsky I., Li J.-P. (2009). Newly Generated Heparanase Knock-Out Mice Unravel Co-Regulation of Heparanase and Matrix Metalloproteinases. PLoS ONE.

[38] Pisano C., Vlodavsky I., Ilan N., Zunino F. (2014). The potential of heparanase as a therapeutic target in cancer. Biochem. Pharmacol..

[39] Rivara S., Milazzo F.M., Giannini G. (2016). Heparanase: A rainbow pharmacological target associated to multiple pathologies including rare diseases. Future Med. Chem..

[40] Vlodavsky I., Ilan N., Naggi A., Casu B. (2007). Heparanase: Structure, Biological Functions, and Inhibition by Heparin-Derived Mimetics of Heparan Sulfate. Curr. Pharm. Des..

[41] Messore A., Madia V.N., Pescatori L., Saccoliti F., Tudino V., De Leo A., Bortolami M., De Vita D., Scipione L., Pepi F. (2018). Novel Symmetrical Benzazolyl Derivatives Endowed with Potent Anti-Heparanase Activity. J. Med. Chem..

[42] Simizu S., Ishida K., Osada H. (2004). Heparanase as a molecular target of cancer chemotherapy. Cancer Sci..

[43] Xu Y.-J., Miao H.-Q., Pan W., Navarro E.C., Tonra J.R., Mitelman S., Camara M.M., Deevi D.S., Kiselyov A.S., Kussie P. (2006). *N*-(4-{[4-(1H-Benzoimidazol-2-yl)-arylamino]-methyl}-phenyl)-benzamide derivatives as small molecule heparanase inhibitors. Bioorganic Med. Chem. Lett..

[44] Pan W., Miao H.-Q., Xu Y.-J., Navarro E.C., Tonra J.R., Corcoran E., Lahiji A., Kussie P., Kiselyov A.S., Wong W.C. (2006). 1-[4-(1*H*-Benzoimidazol-2-yl)-phenyl]-3-[4-(1*H*-benzoimidazol-2-yl)-phenyl]-urea derivatives as small molecule heparanase inhibitors. Bioorganic Med. Chem. Lett..

[45] Torre L.A., Bray F., Siegel R.L., Ferlay J., Lortet-Tieulent J., Jemal A. (2015). Global cancer statistics, 2012: Global Cancer Statistics, 2012. CA Cancer J. Clin..

[46] Marino S., Roodman G.D. (2018). Multiple Myeloma and Bone: The Fatal Interaction. Cold Spring Harb. Perspect. Med..

[47] Purushothaman A., Sanderson R.D. (2020). Heparanase: A Dynamic Promoter of Myeloma Progression. Adv. Exp. Med. Biol..

[48] Wu L., Davies G.J. (2020). An Overview of the Structure, Mechanism and Specificity of Human Heparanase. Adv. Exp. Med. Biol..

[49] Wu L., Viola C.M., Brzozowski A.M., Davies G.J. (2015). Structural characterization of human heparanase reveals insights into substrate recognition. Nat. Struct. Mol. Biol..

[50] Wu L., Jiang J., Jin Y., Kallemeijn W.W., Kuo C.-L., Artola M., Dai W., Van Elk C., van Eijk M., Van Der Marel G.A. (2017). Activity-based probes for functional interrogation of retaining β-glucuronidases. Nat. Chem. Biol..

[51] Khamaysi I., Hamo-Giladi D.B., Abassi Z. (2020). Heparanase in Acute Pancreatitis. Adv. Exp. Med. Biol..

[52] Lerner I., Hermano E., Zcharia E., Rodkin D., Bulvik R., Doviner V., Rubinstein A.M., Ishai-Michaeli R., Atzmon R., Sherman Y. (2011). Heparanase powers a chronic inflammatory circuit that promotes colitis-associated tumorigenesis in mice. J. Clin. Investig..

[53] Masola V., Gambaro G., Onisto M. (2020). Impact of Heparanse on Organ Fibrosis. Adv. Exp. Med. Biol..

[54] Abassi Z., Goligorsky M.S. (2020). Heparanase in Acute Kidney Injury. Adv. Exp. Med. Biol..

[55] Van Der Vlag J., Buijsers B. (2020). Heparanase in Kidney Disease. Adv. Exp. Med. Biol..

[56] Rabelink T., Berg B.M.V.D., Garsen M., Wang G., Elkin M., Van Der Vlag J. (2017). Heparanase: Roles in cell survival, extracellular matrix remodelling and the development of kidney disease. Nat. Rev. Nephrol..

[57] Gil N., Goldberg R., Neuman T., Garsen M., Zcharia E., Rubinstein A.M., Van Kuppevelt T., Meirovitz A., Pisano C., Li J.-P. (2011). Heparanase Is Essential for the Development of Diabetic Nephropathy in Mice. Diabetes.

[58] Simeonovic C.J., Popp S.K., Brown D.J., Li F.-J., Lafferty A.R.A., Freeman C., Parish C.R. (2020). Heparanase and Type 1 Diabetes. Adv. Exp. Med. Biol..

[59] Shi C., Wang C., Wang H., Yang C., Cai F., Zeng F., Cheng F., Liu Y., Zhou T., Deng B. (2020). The Potential of Low Molecular Weight Heparin to Mitigate Cytokine Storm in Severe COVID-19 Patients: A Retrospective Cohort Study. Clin. Transl. Sci..

[60] Buijsers B., Yanginlar C., Maciej-Hulme M.L., de Mast Q., van der Vlag J. (2020). Beneficial non-anticoagulant mechanisms underlying heparin treatment of COVID-19 patients. EBioMedicine.

[61] Martino C., Kellman B.P., Sandoval D.R., Clausen T.M., Marotz C.A., Song S.J., Wandro S., Zaramela L.S., Salido Benítez R.A., Zhu Q. (2020). Bacterial modification of the host glycosaminoglycan heparan sulfate modulates SARS-CoV-2 infectivity. bioRxiv.

[62] Agelidis A., Shukla D. (2020). Heparanase, Heparan Sulfate and Viral Infection. Adv. Exp. Med. Biol..

