# Supplementary material for: New Heparanase-Inhibiting Triazolo-Thiadiazoles Attenuate Primary Tumor Growth and Metastasis
Source: Cancers (Basel). 2021 Jun 13;13(12):2959. doi: 10.3390/cancers13122959 (PMC8231572; doi:10.3390/cancers13122959)
Supplement: Supplementary file 1 [file cancers-13-02959-s001.zip › cancers-1218559-supplementary.pdf]

Supplementary Materials: New Heparanase-Inhibiting Triazolo-Thiadiazoles Attenuate Primary Tumor Growth and Metastasis

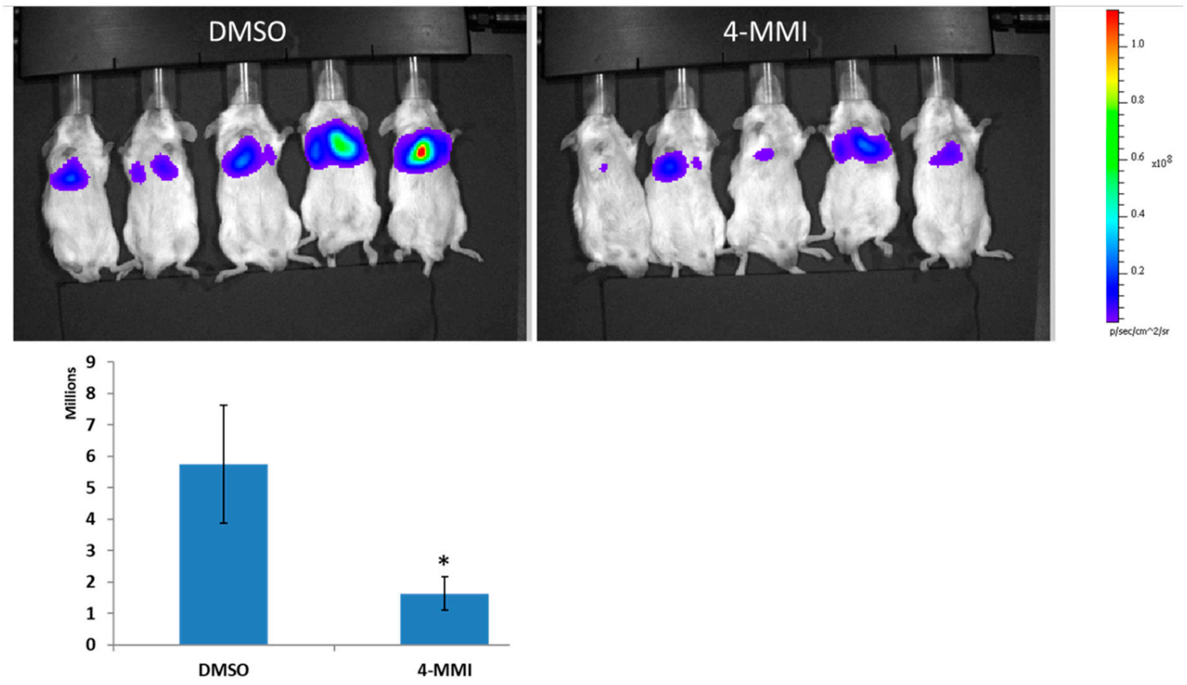

Figure S1. Inhibition of 4T1 breast carcinoma experimental metastasis. Luciferase-labeled 4T1 breast carcinoma cells were injected i.v into Balb/c mice (n=6). Vehicle (DMSO alone) or 4-MMI (500 μg/mouse) were injected (i.p; 0.1 ml/mouse) 20 min prior to cell inoculation. Metastasis was inspected by IVIS on day 11 after cell inoculation. Quantification of the luciferase intensities is shown graphically in the lower panel. For more detail see figure 4. (* = 0.048).

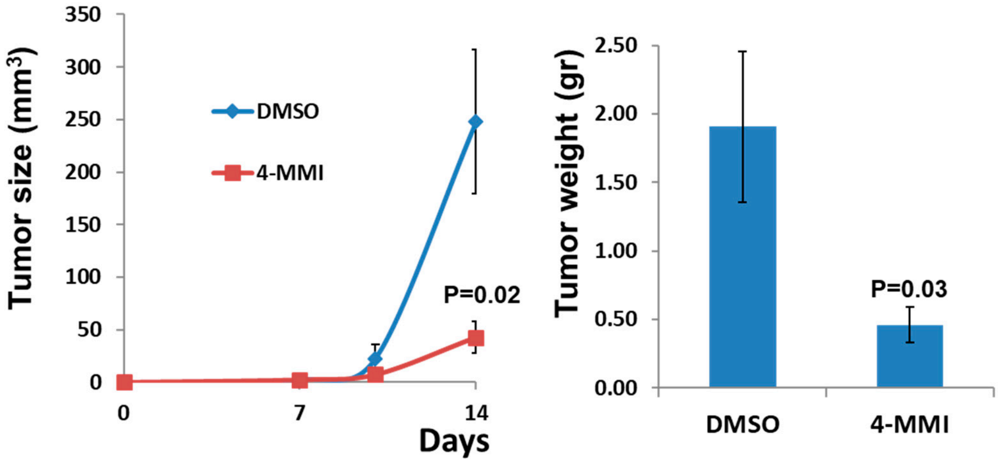

Figure S2. MPC-11 tumor growth. MPC-11 cells were inoculated subcutaneously at the right flank of 6-weeks old female Balb/C mice (n=6). Mice were treated with either DMSO (0.1 ml/mouse) or 4-MMI (daily 200 µg/mouse, i.p.) starting on day 2 after the injection of tumor cells, for 12 days. Tumor size was determined on days 8 and 12 (left panel). At the end of the experiment, mice were sacrificed and the tumors were resected and weighed (right panel). For more detail see figure 5.

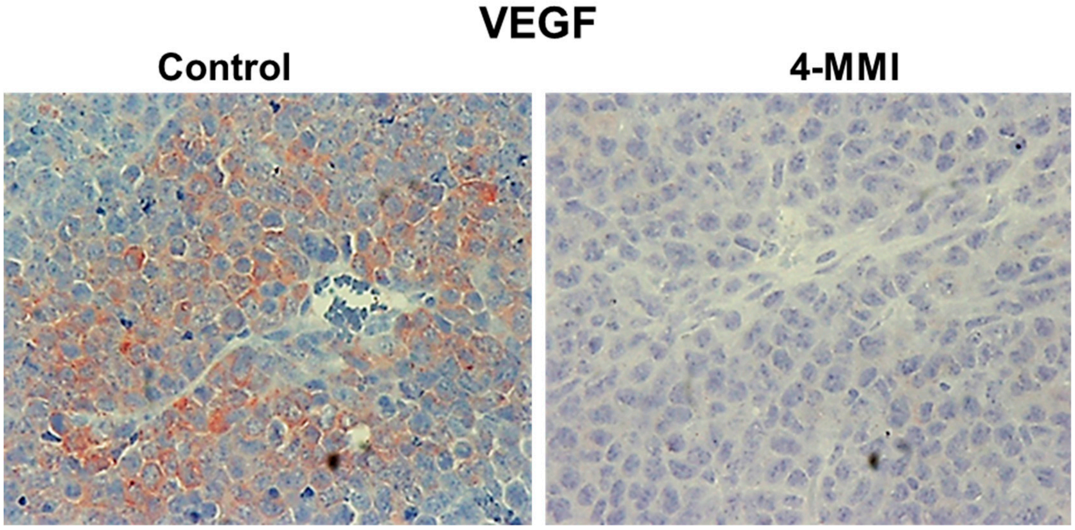

Figure S3. Immunostaining. Tumors produced by untreated (control) and 4-MMI treated Balb/c mice were removed (day 13), fixed and embedded in paraffin. Five micron sections were subjected to immunostaining applying anti-VEGF antibodies. Shown are representative images (original magnification x100). No immunostaining was observed when the primary antibody was omitted or when purified VEGF protein (1 µg/ml) was added together with the primary antibody.

Supplementary methods

Immunostaining. Immunostaining of formalin-fixed, paraffin-embedded 5-micron sections was performed essentially as described [30–32], utilizing anti-VEGF polyclonal antibody (Santa Cruz Biotechnology, A-20 sc-152). Briefly, slides were deparaffinized, rehydrated and endogenous peroxidase activity was quenched (30 min) by 3% hydrogen peroxide in methanol. Slides were then

subjected to antigen retrieval (boiling for 20 min), blocking (10% normal goat serum) and incubation (20 h, 4oC) with anti-VEGF antibody [27-29] diluted (1: 100) in blocking solution. Slides were extensively washed with PBS, incubated with a secondary reagent (Envision kit) and color was developed with the AEC reagent (Dako, Glostrup, Denmark) [63-65].

Mass, ^{1}H and ^{13}C NMR spectra:

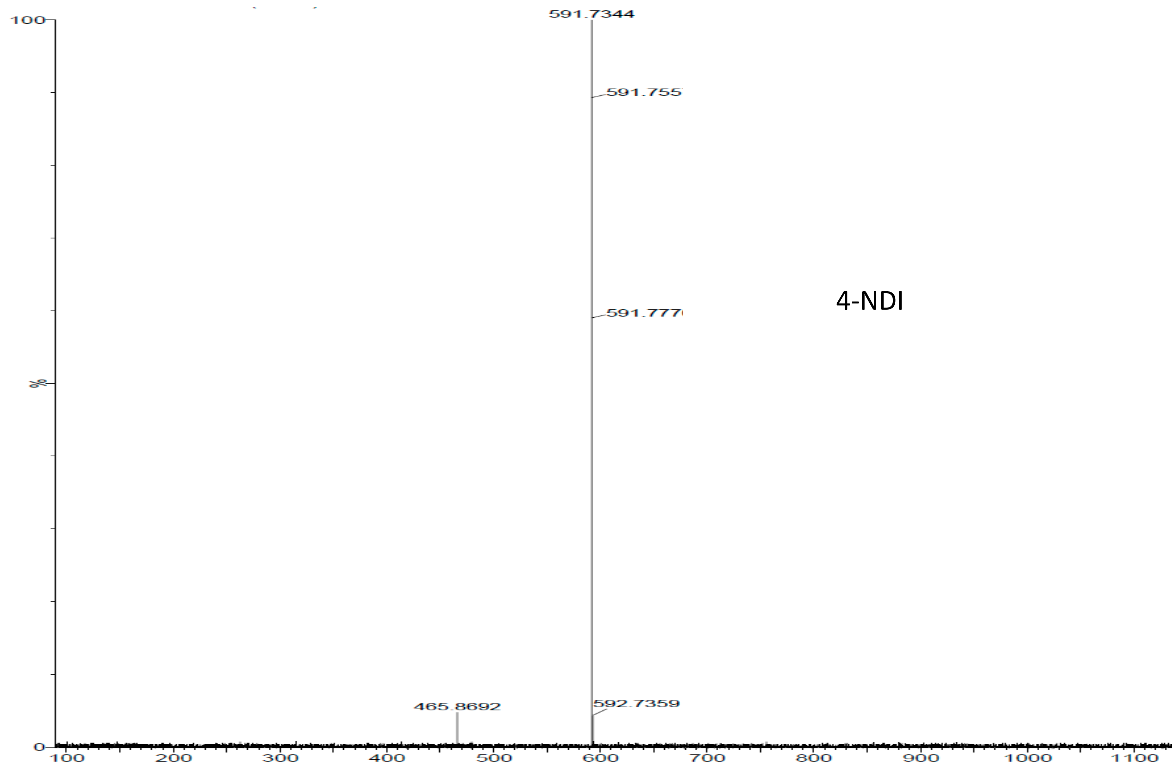

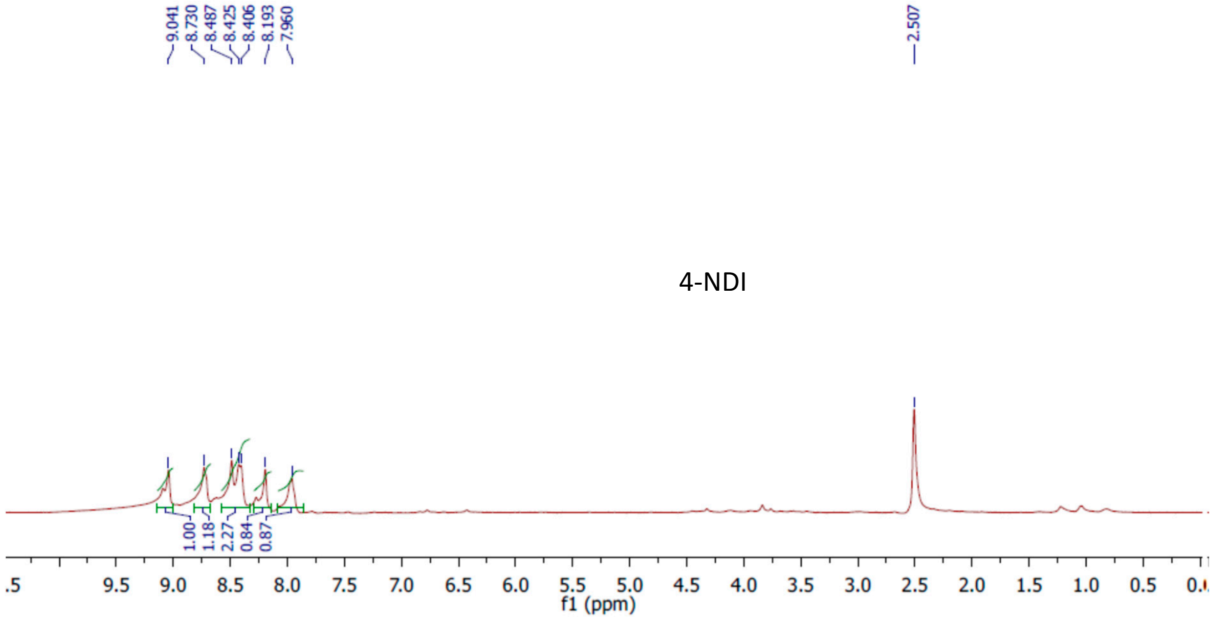

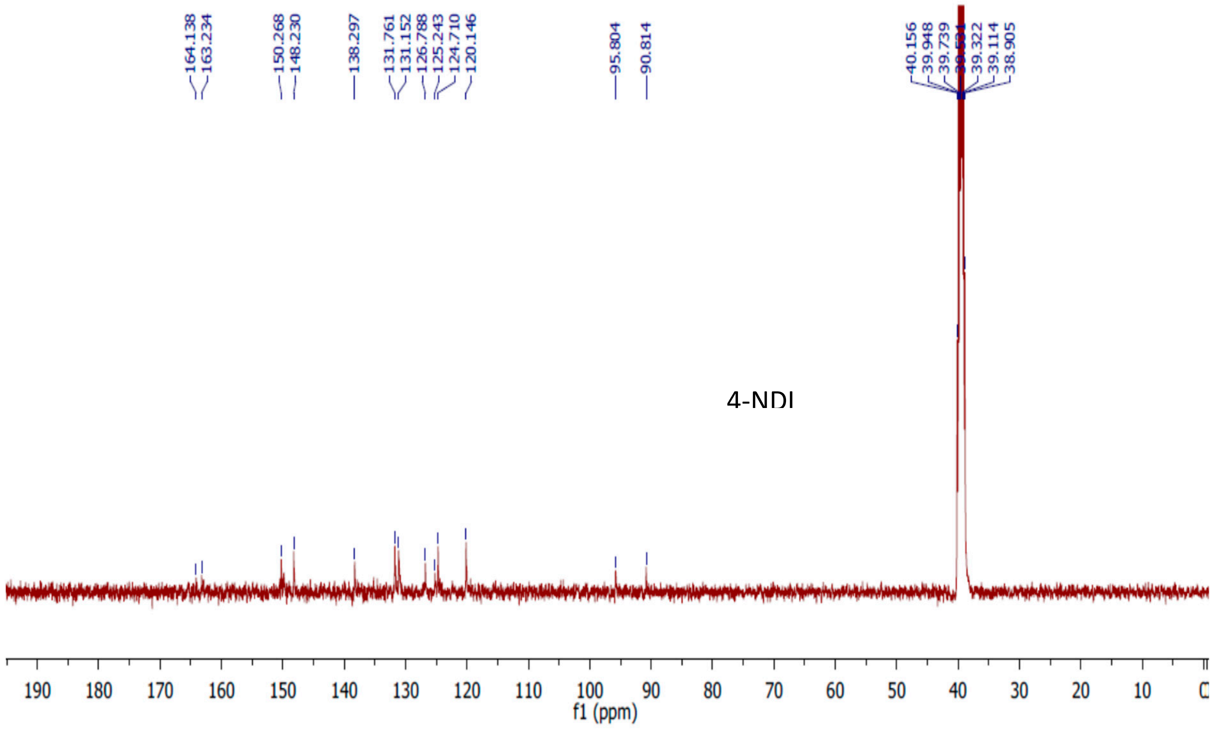
164.138
163.234
150.268
148.230
138.297
131.761
131.152
126.788
125.243
124.710
120.146
95.804
90.814
40.156
39.948
39.739
39.322
39.114
38.905
4-NDI
190 180 170 160 150 140 130 120 110 100 90 80 70 60 50 40 30 20 10
f1 (ppm)

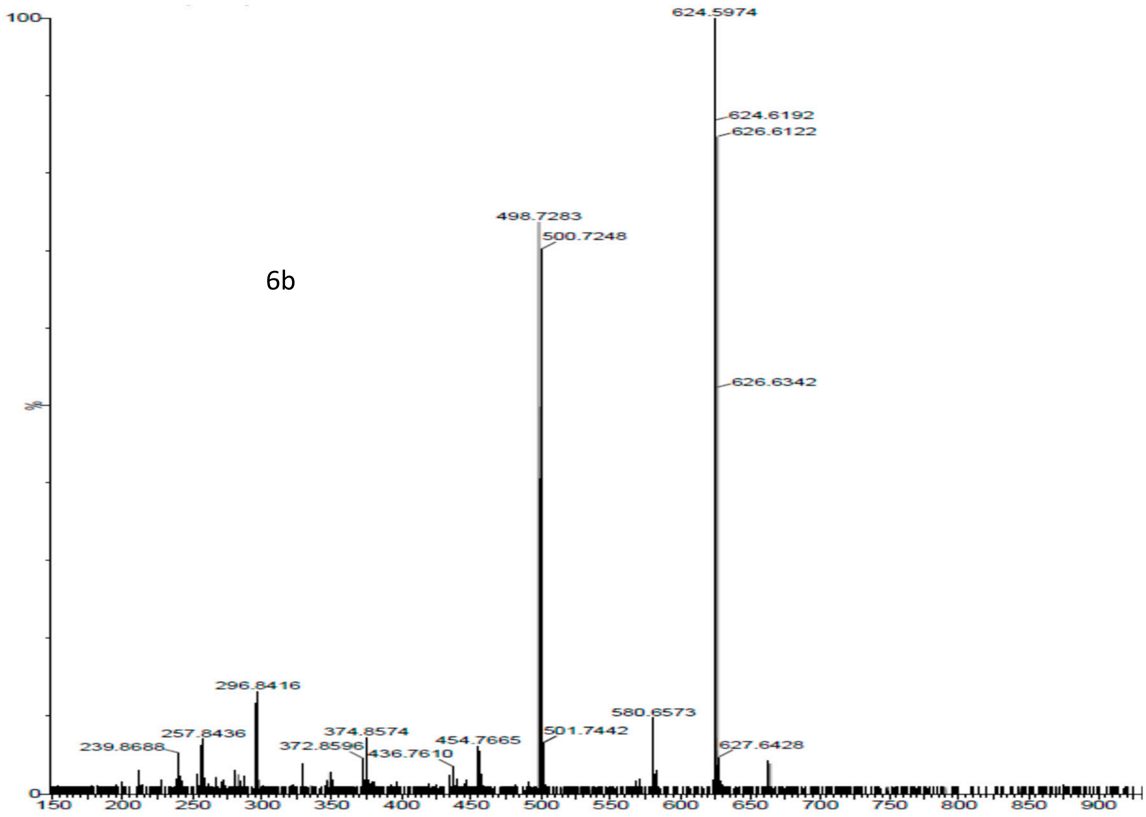
100
%
0
624.5974
624.6192
626.6122
626.6342
498.7283
500.7248
6b
296.8416
239.8688
257.8436
374.8574
372.8596
436.7610
454.7665
501.7442
580.6573
627.6428
150 200 250 300 350 400 450 500 550 600 650 700 750 800 850 900

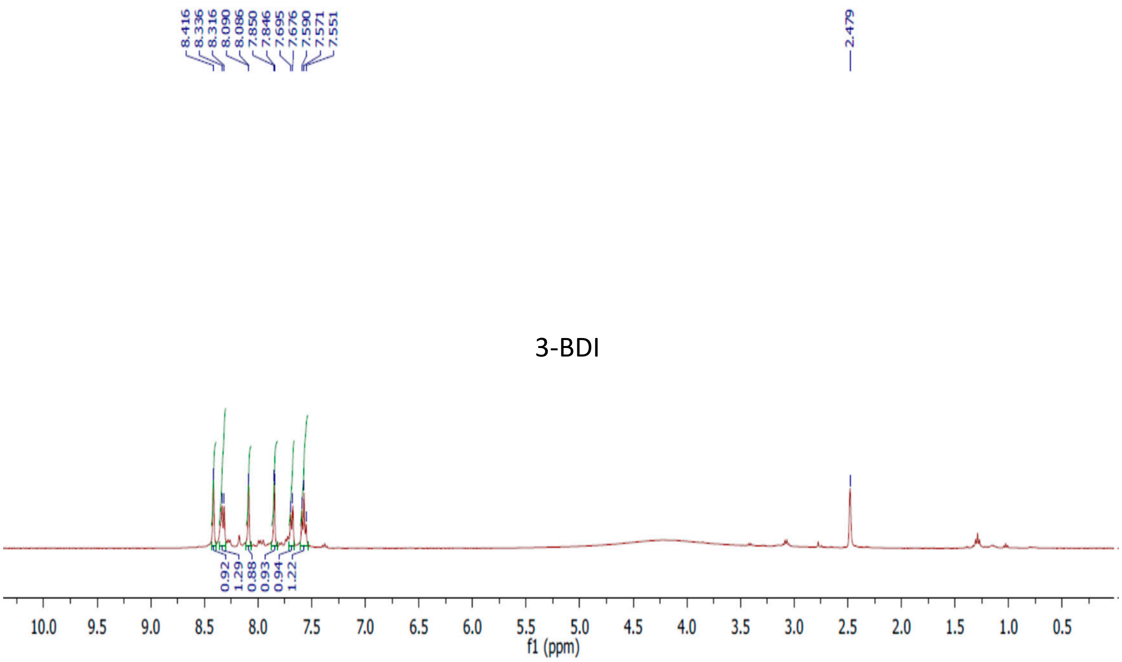
8.416
8.336
8.316
8.090
8.086
7.850
7.846
7.695
7.676
7.590
7.571
7.551
2.479
3-BDI
0.92
1.29
0.88
0.93
0.94
1.22
f1 (ppm)

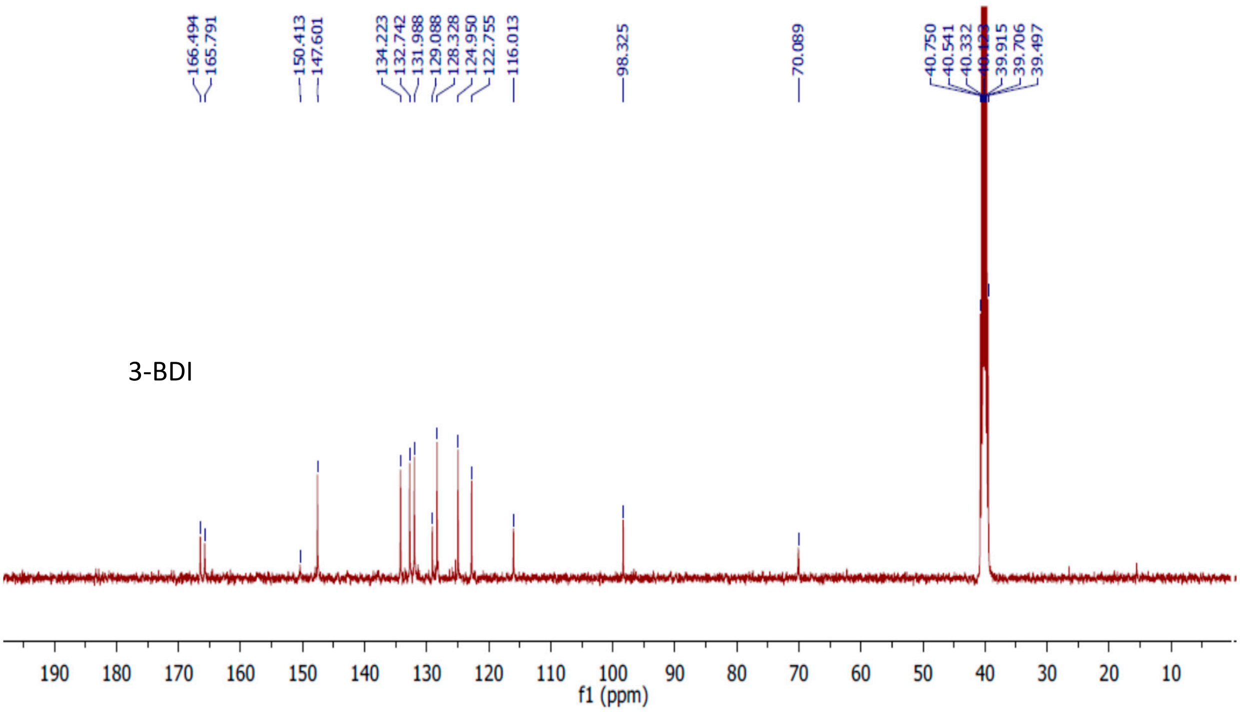
166.494
165.791
150.413
147.601
134.223
132.742
131.988
129.088
128.328
124.950
122.755
116.013
98.325
70.089
40.750
40.541
40.332
39.915
39.706
39.497
3-BDI
f1 (ppm)

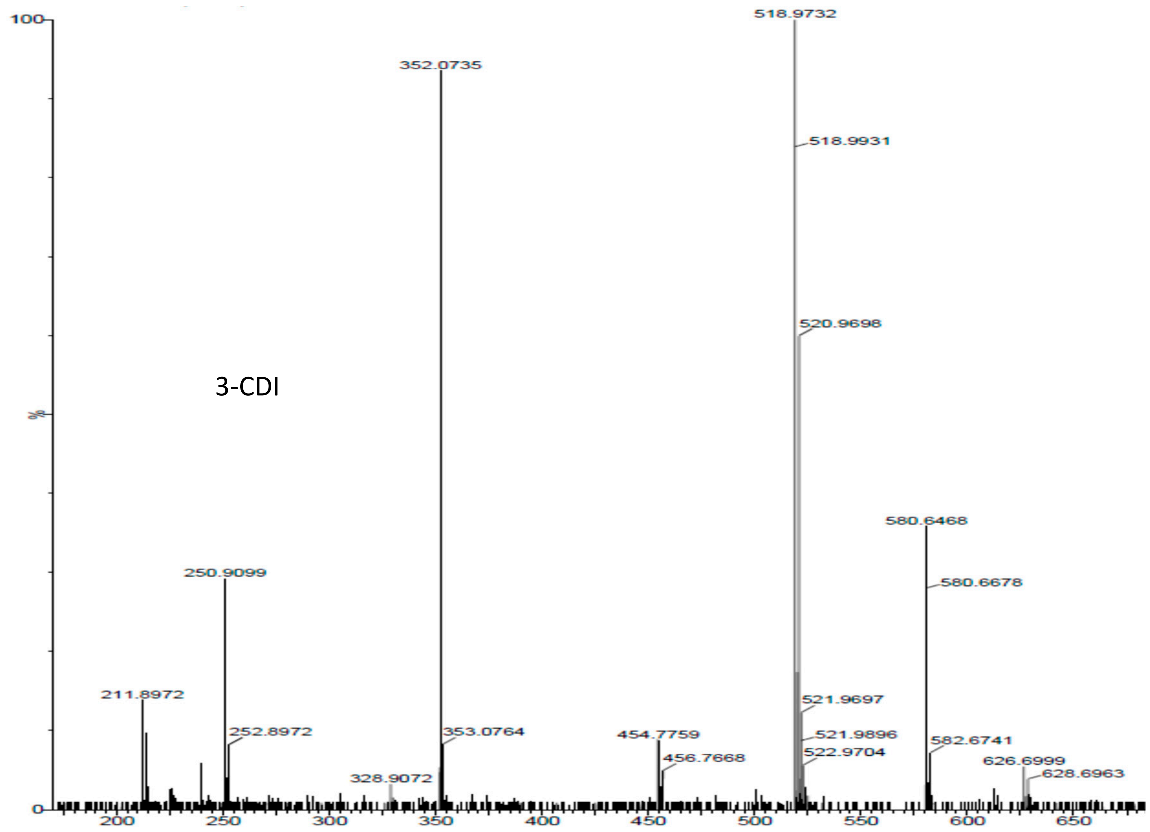
3-CDI
518.9732
518.9931
520.9698
521.9697
521.9896
522.9704
352.0735
353.0764
580.6468
580.6678
582.6741
250.9099
252.8972
211.8972
328.9072
454.7759
456.7668
626.6999
628.6963
100
%
0
200 250 300 350 400 450 500 550 600 650

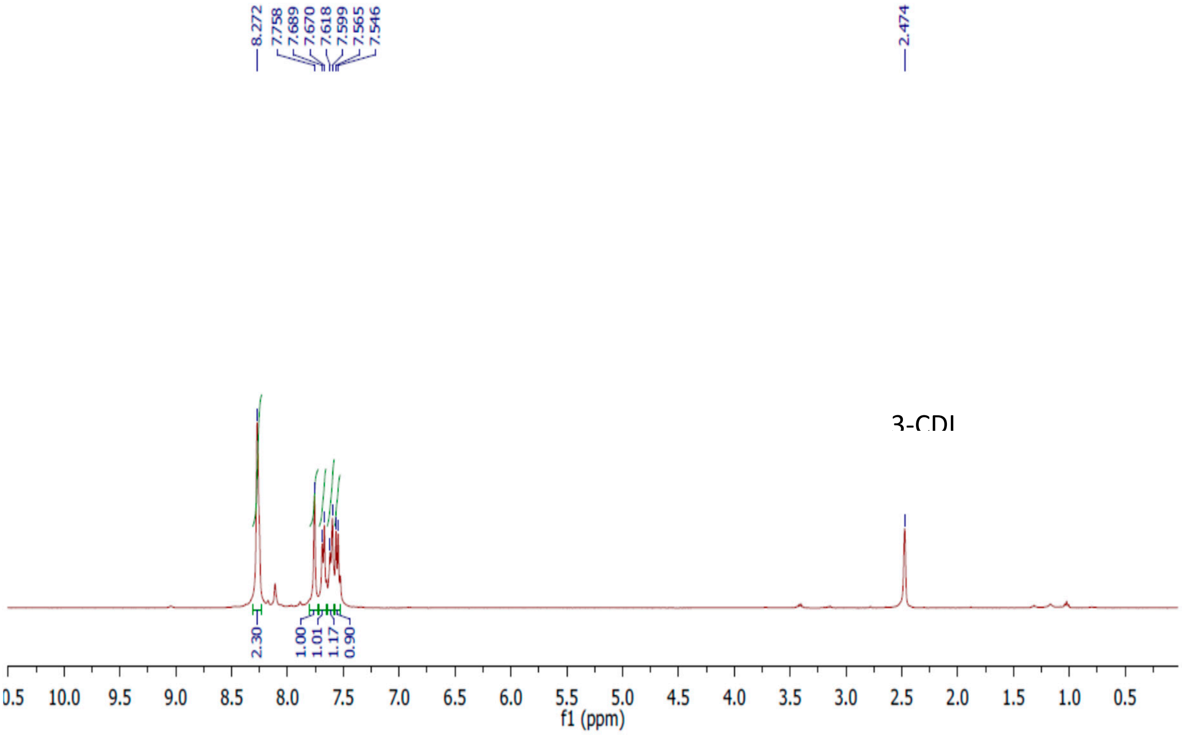
8.272
7.758
7.689
7.670
7.618
7.599
7.565
7.546
2.474
3-CDI
2.30
1.00
1.01
1.17
0.90
f1 (ppm)

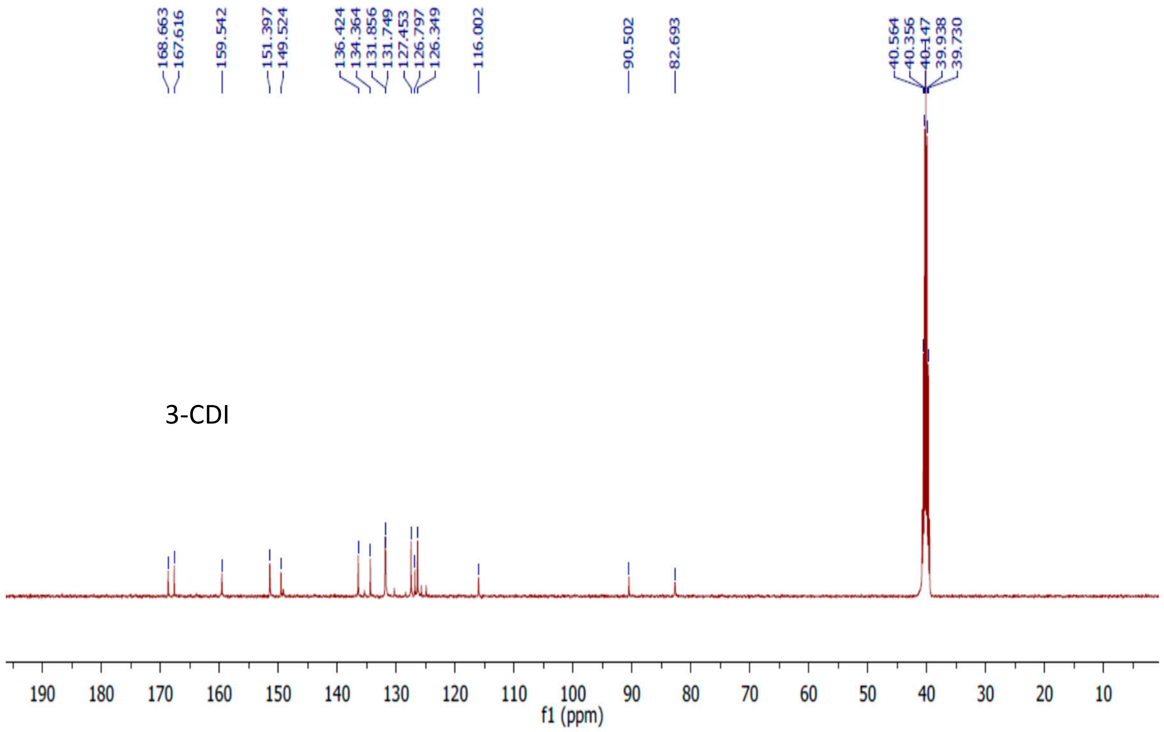
168.663
167.616
159.542
151.397
149.524
136.424
134.364
131.856
131.749
127.453
126.797
126.349
116.002
90.502
82.693
40.564
40.356
40.147
39.938
39.730
3-CDI
190 180 170 160 150 140 130 120 110 100 90 80 70 60 50 40 30 20 10
f1 (ppm)

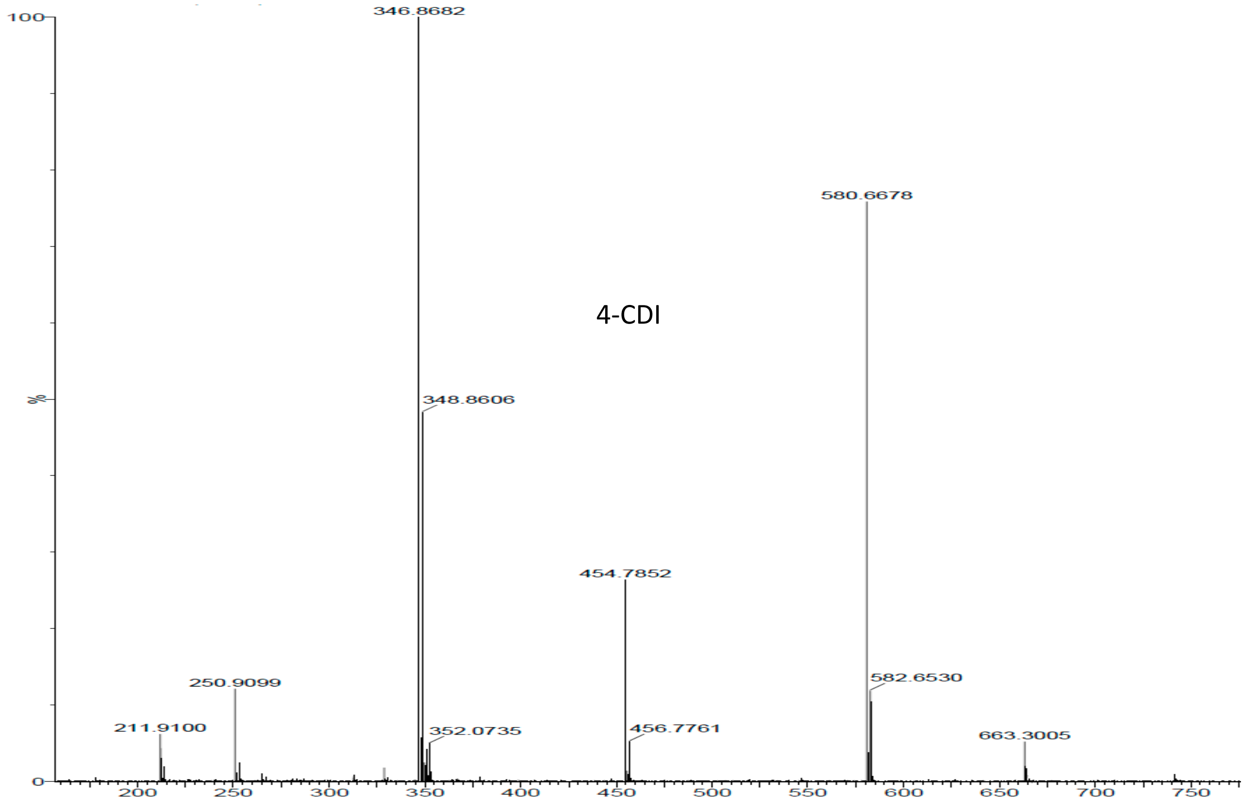
100
%
0
346.8682
348.8606
352.0735
580.6678
582.6530
454.7852
456.7761
250.9099
211.9100
663.3005
4-CDI
200 250 300 350 400 450 500 550 600 650 700 750

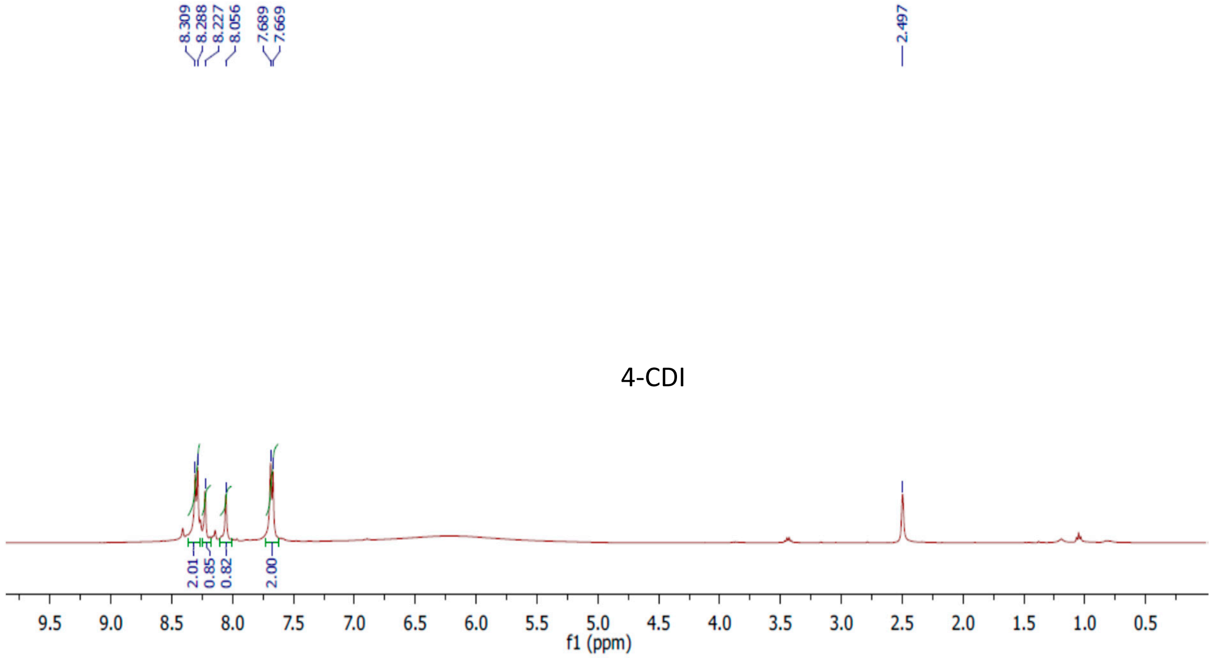

8.309
8.288
8.227
8.056
7.689
7.669
2.497
4-CDI
2.01
0.85
0.82
2.00
9.5 9.0 8.5 8.0 7.5 7.0 6.5 6.0 5.5 5.0 4.5 4.0 3.5 3.0 2.5 2.0 1.5 1.0 0.5
f1 (ppm)

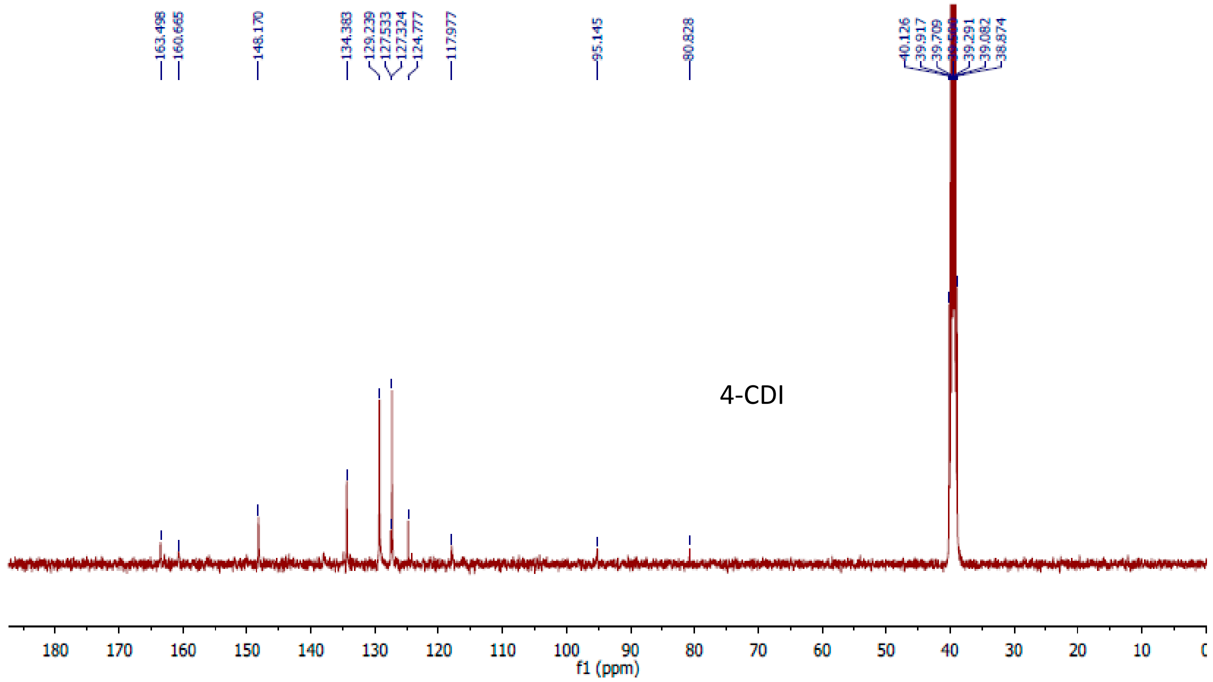

163.498
160.665
148.170
134.383
129.239
127.533
127.324
124.777
117.977
95.145
80.828
40.126
39.917
39.709
39.291
39.082
38.874
4-CDI
180 170 160 150 140 130 120 110 100 90 80 70 60 50 40 30 20 10
f1 (ppm)

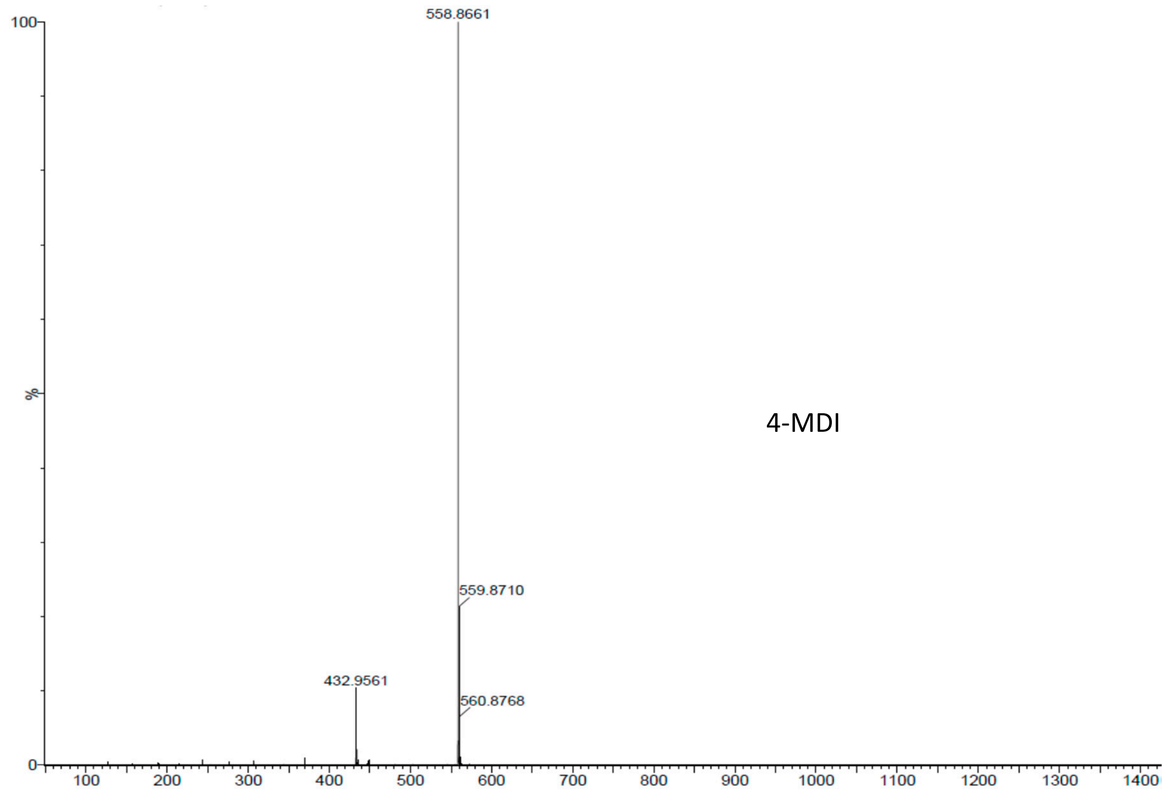
558.8661
559.8710
560.8768
432.9561
100
%
0
100
200
300
400
500
600
700
800
900
1000
1100
1200
1300
1400
4-MDI

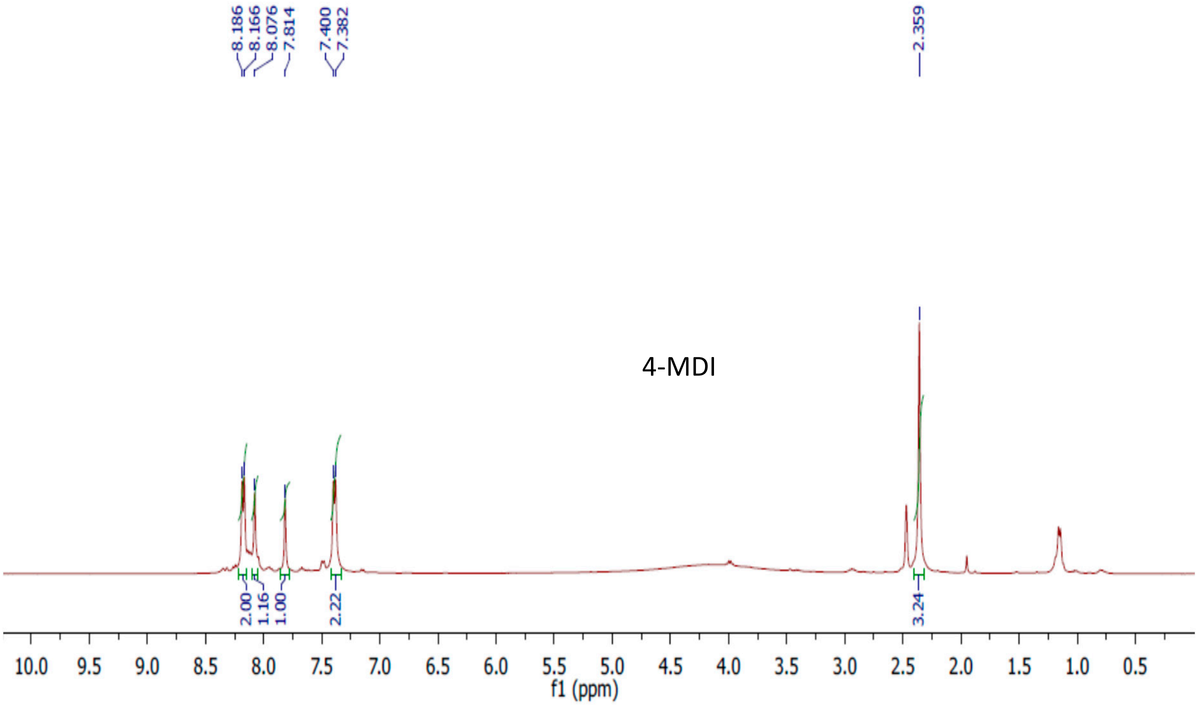
8.186
8.166
8.076
7.814
7.400
7.382
2.359
4-MDI
2.00
1.16
1.00
2.22
3.24
10.0
9.5
9.0
8.5
8.0
7.5
7.0
6.5
6.0
5.5
5.0
4.5
4.0
3.5
3.0
2.5
2.0
1.5
1.0
0.5
f1 (ppm)

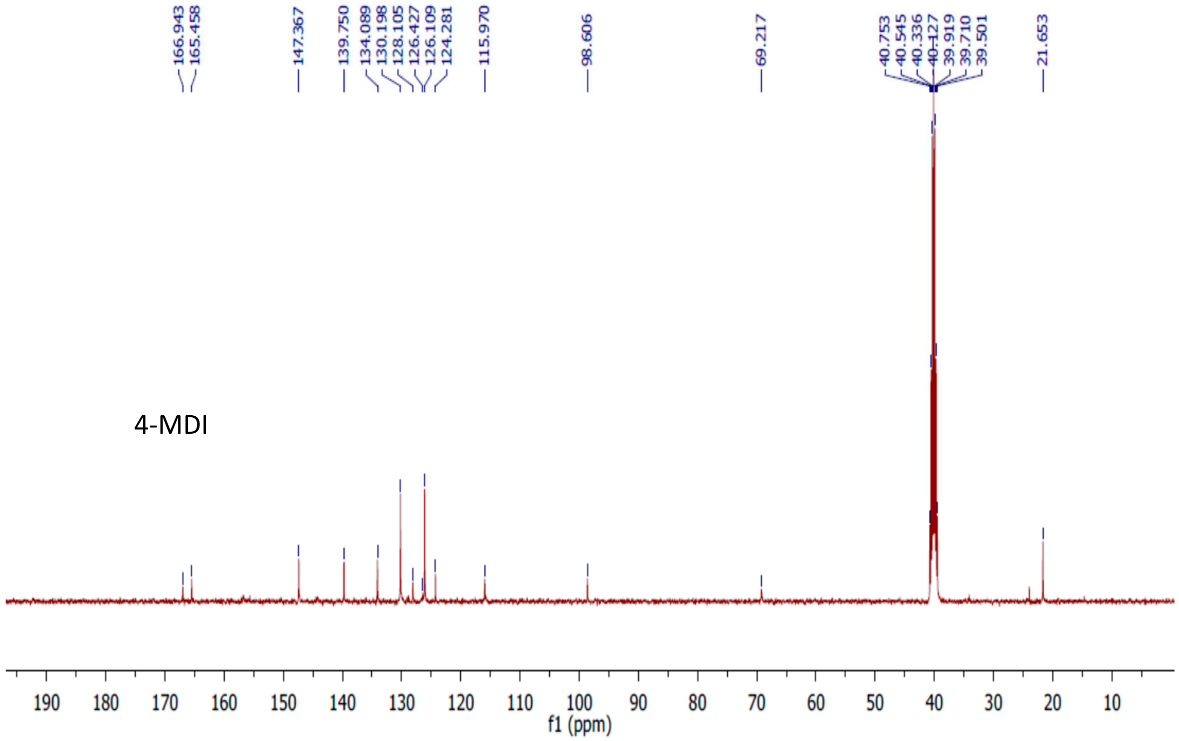
166.943
165.458
147.367
139.750
134.089
130.198
128.105
126.427
126.109
124.281
115.970
98.606
69.217
40.753
40.545
40.336
40.127
39.919
39.710
39.501
21.653
4-MDI
190 180 170 160 150 140 130 120 110 100 90 80 70 60 50 40 30 20 10
f1 (ppm)

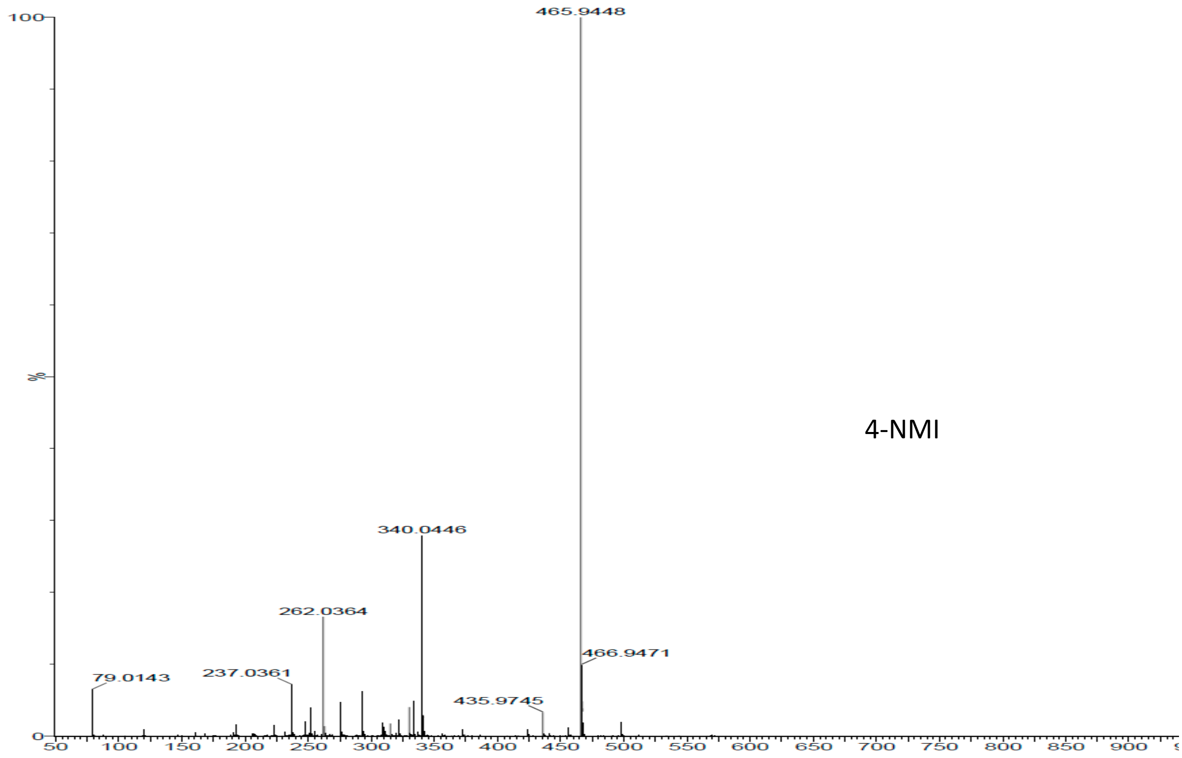
465.9448
100
%
0
4-NMI
340.0446
262.0364
466.9471
79.0143
237.0361
435.9745
50 100 150 200 250 300 350 400 450 500 550 600 650 700 750 800 850 900

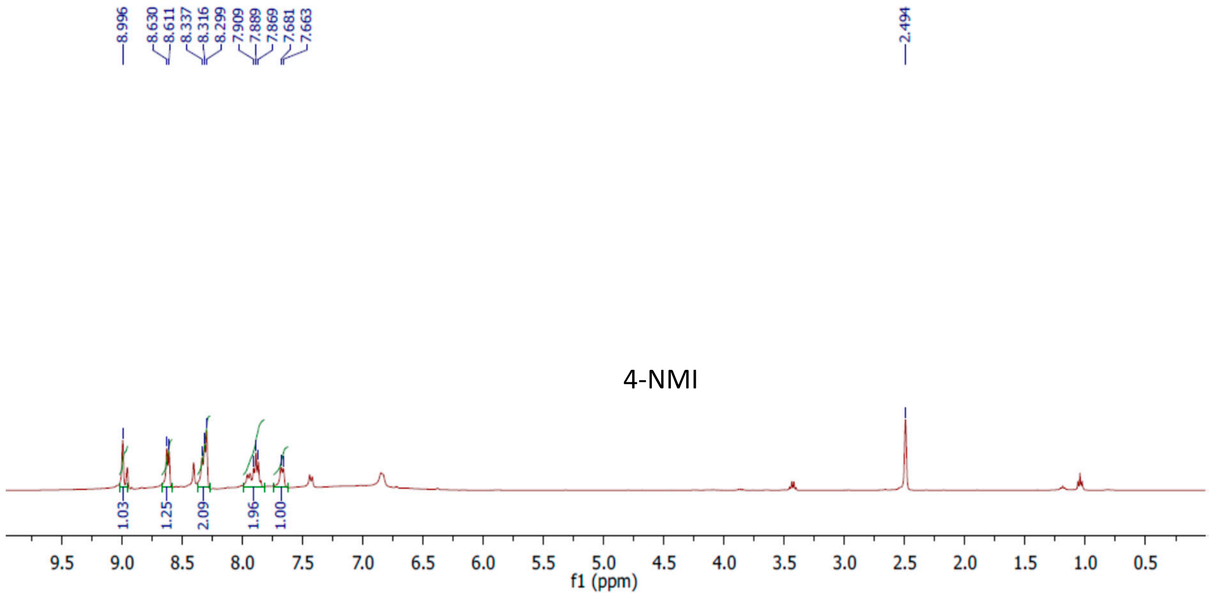
8.996
8.630
8.611
8.337
8.316
8.299
7.909
7.889
7.869
7.681
7.663
2.494
4-NMI
1.03
1.25
2.09
1.96
1.00
f1 (ppm)

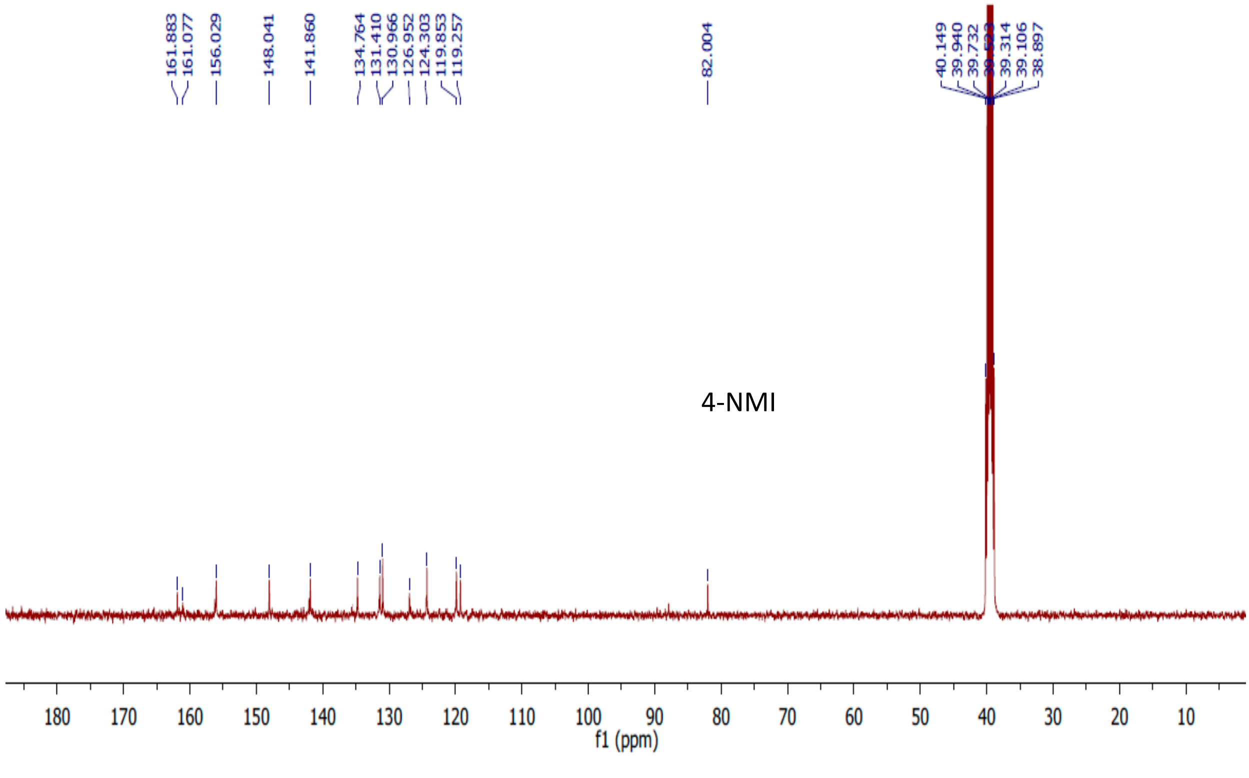
161.883
161.077
156.029
148.041
141.860
134.764
131.410
130.966
126.952
124.303
119.853
119.257
82.004
40.149
39.940
39.732
39.314
39.106
38.897
4-NMI
f1 (ppm)

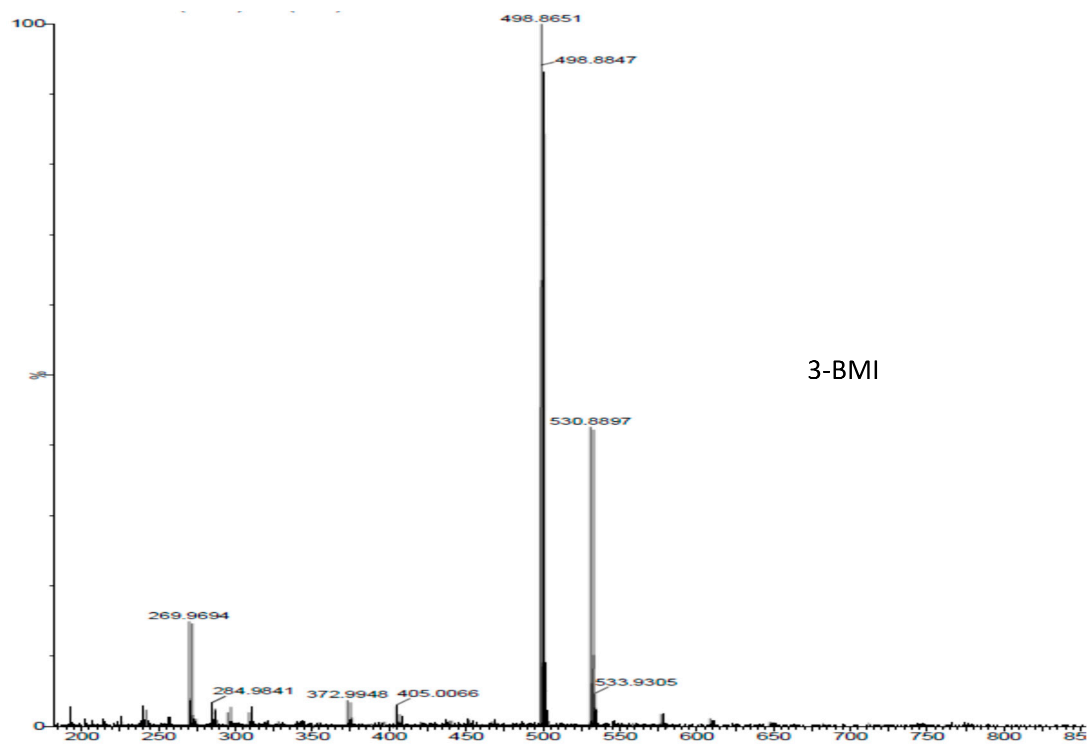
3-BMI
100
%
0
498.8651
498.8847
530.8897
533.9305
269.9694
284.9841
372.9948
405.0066
200 250 300 350 400 450 500 550 600 650 700 750 800 85

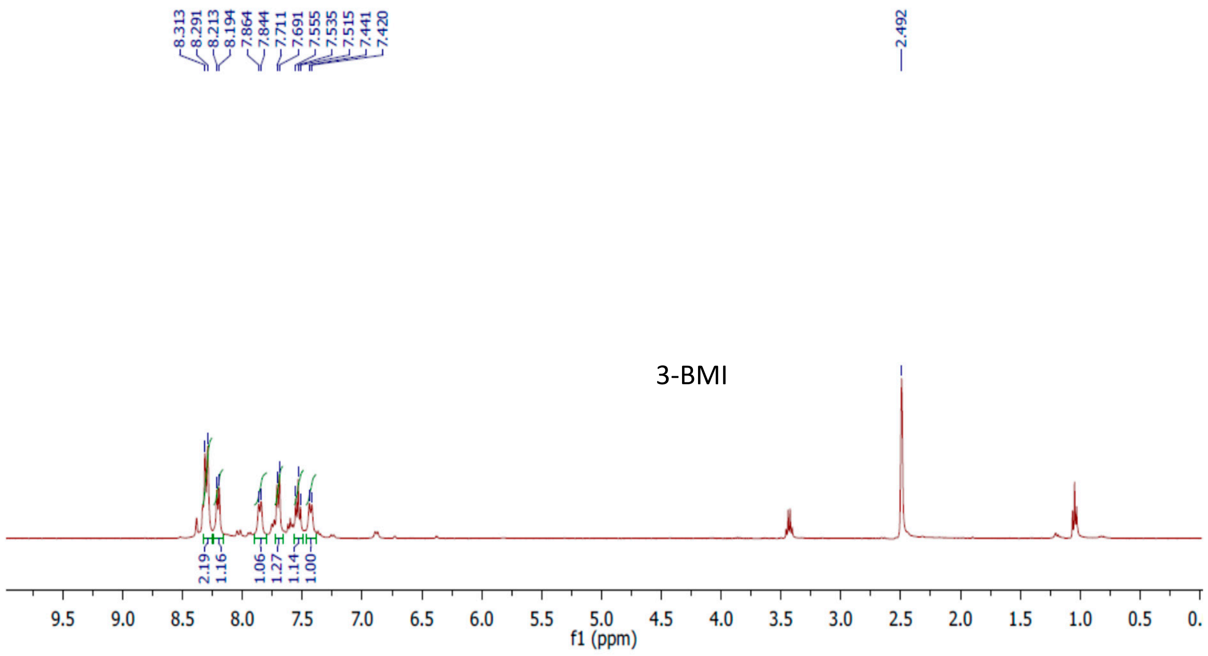
3-BMI
8.313
8.291
8.213
8.194
7.864
7.844
7.711
7.691
7.555
7.535
7.515
7.441
7.420
2.492
2.19
1.16
1.06
1.27
1.14
1.00
9.5 9.0 8.5 8.0 7.5 7.0 6.5 6.0 5.5 5.0 4.5 4.0 3.5 3.0 2.5 2.0 1.5 1.0 0.5 0.
f1 (ppm)

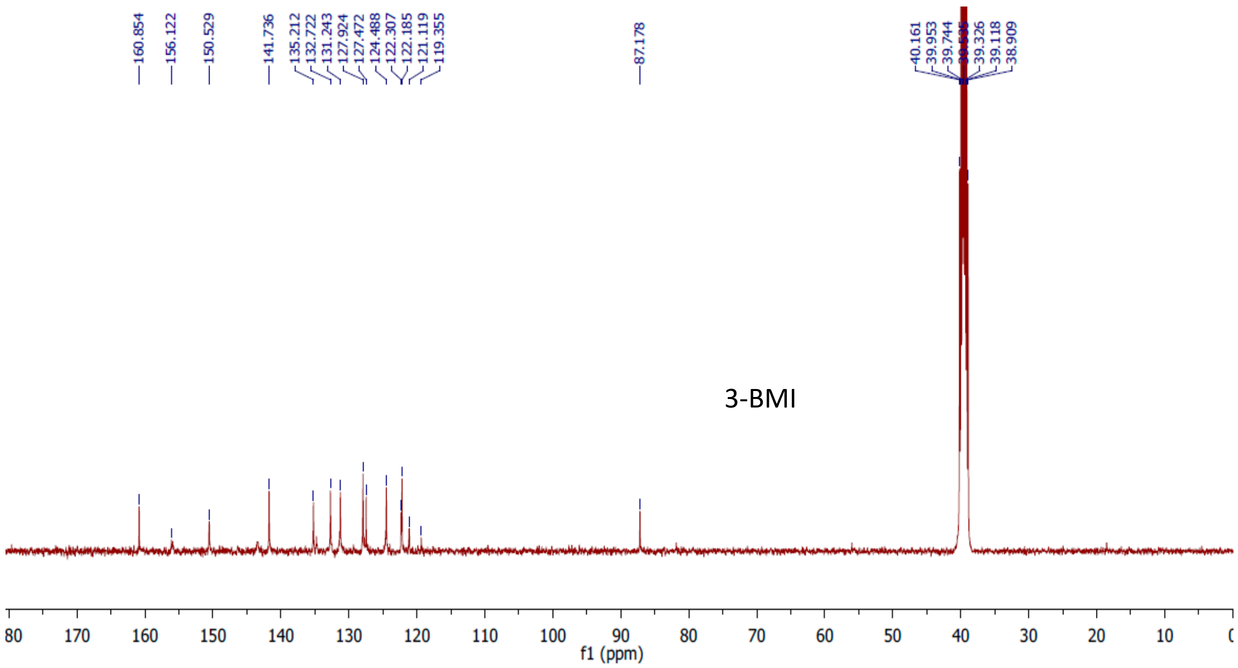

160.854
156.122
150.529
141.736
135.212
132.722
131.243
127.924
127.472
124.488
122.307
122.185
121.119
119.355
87.178
40.161
39.953
39.744
39.326
39.118
38.909
f1 (ppm)

3-BMI

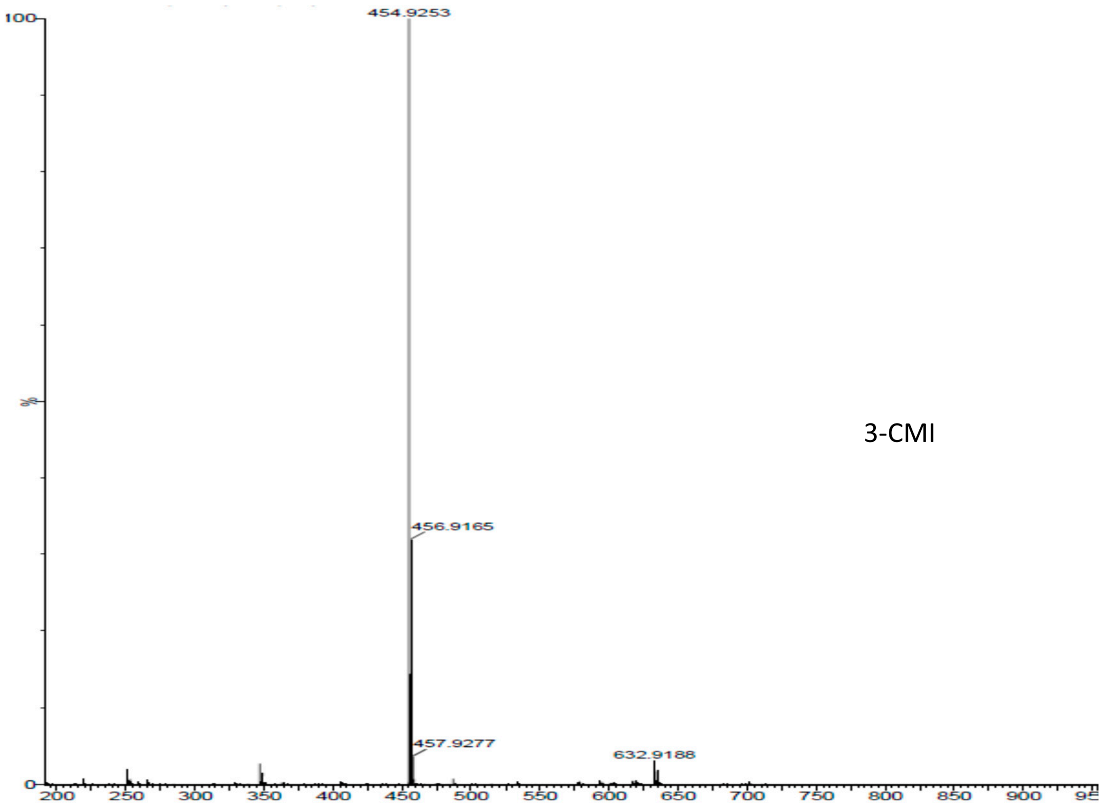

100
%
0
454.9253
456.9165
457.9277
632.9188

3-CMI

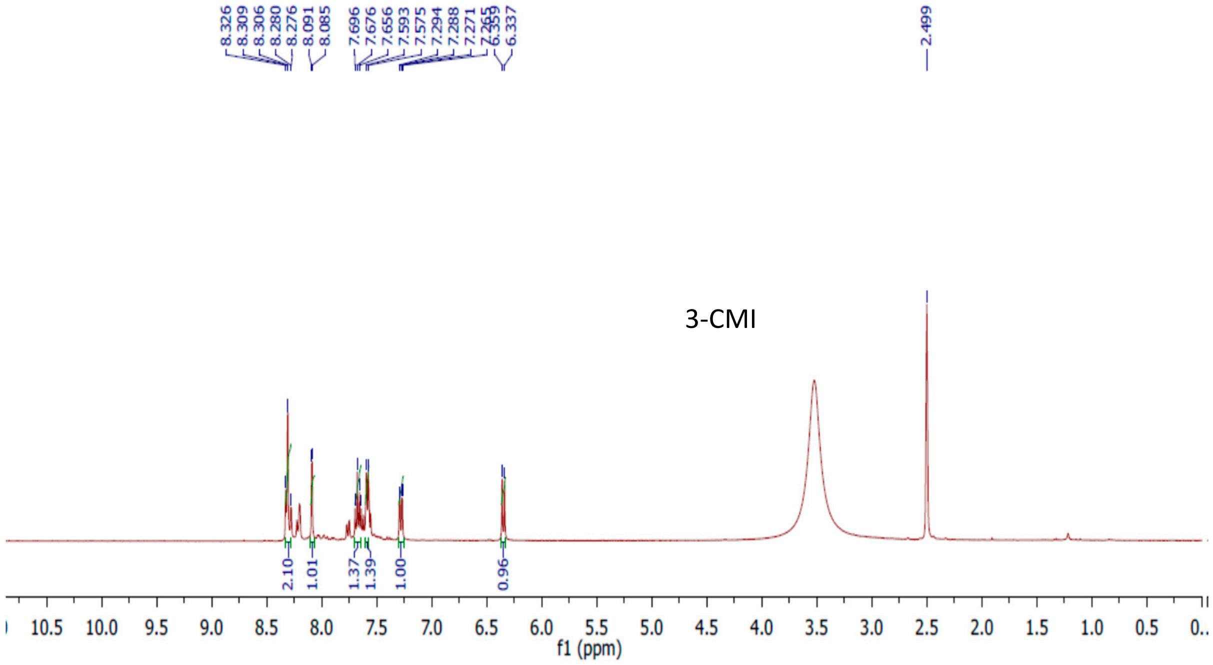
8.326
8.309
8.306
8.280
8.276
8.091
8.085
7.696
7.676
7.656
7.593
7.575
7.294
7.288
7.271
7.265
6.359
6.337
2.499
3-CMI
2.10
1.01
1.37
1.39
1.00
0.96
10.5 10.0 9.5 9.0 8.5 8.0 7.5 7.0 6.5 6.0 5.5 5.0 4.5 4.0 3.5 3.0 2.5 2.0 1.5 1.0 0.5
f1 (ppm)

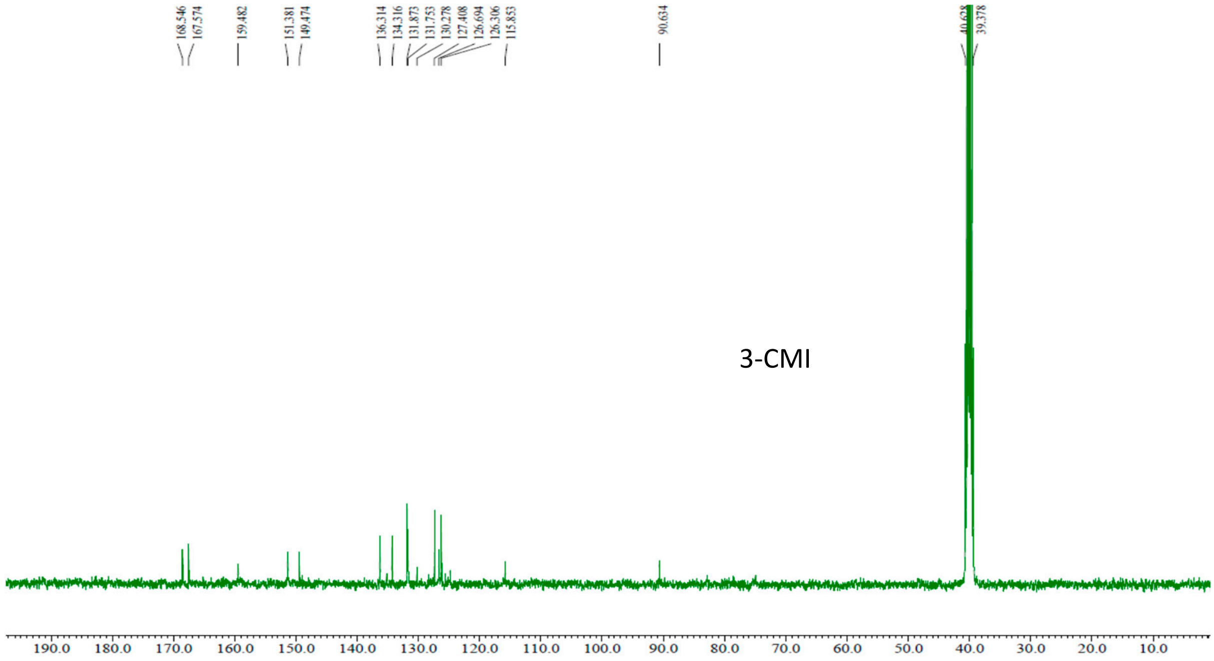
168.546
167.574
159.482
151.381
149.474
136.314
134.316
131.873
131.753
130.278
127.408
126.694
126.306
115.853
90.634
40.628
39.378
3-CMI
190.0 180.0 170.0 160.0 150.0 140.0 130.0 120.0 110.0 100.0 90.0 80.0 70.0 60.0 50.0 40.0 30.0 20.0 10.0

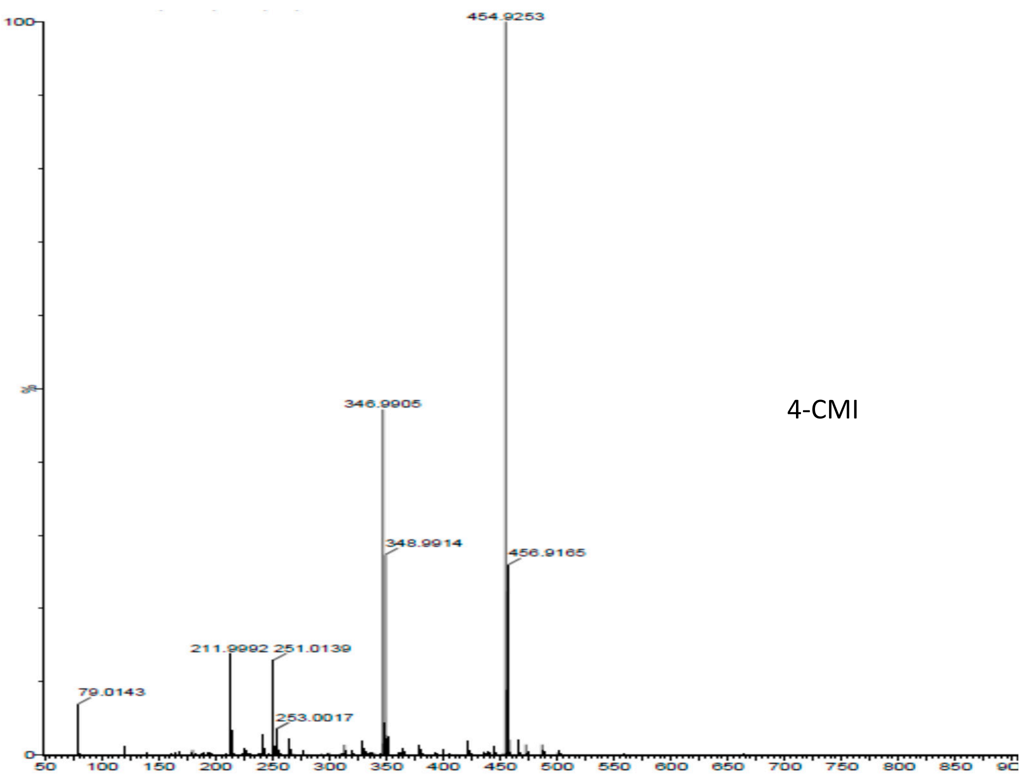

454.9253
346.9905
348.9914
456.9165
211.9992
251.0139
79.0143
253.0017
4-CMI

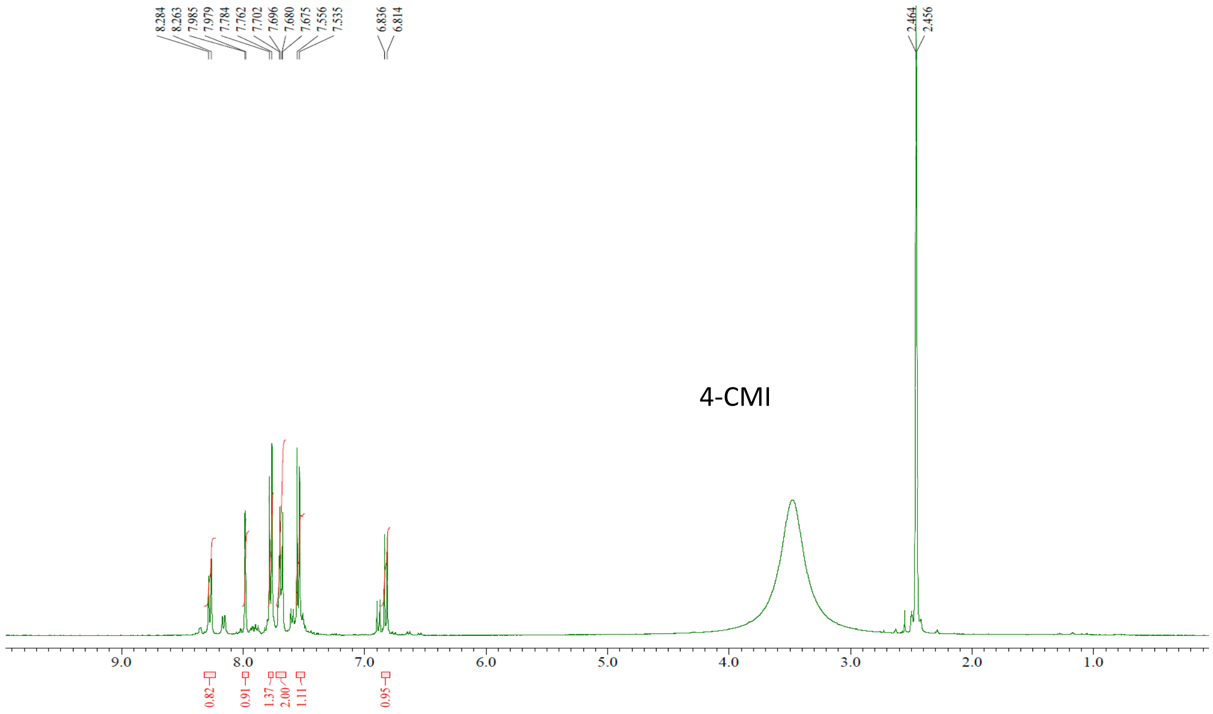

8.284
8.263
7.985
7.979
7.784
7.762
7.702
7.696
7.680
7.675
7.556
7.535
6.836
6.814
2.464
2.456
4-CMI
0.82
0.91
1.37
2.00
1.11
0.95

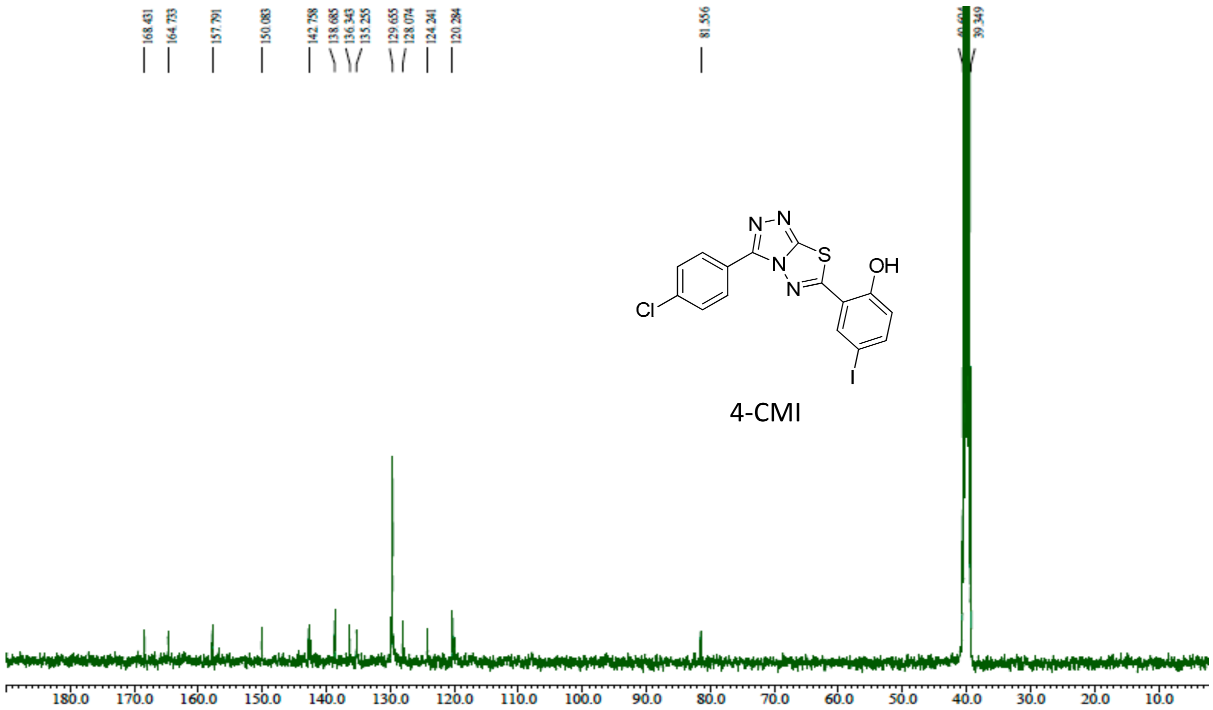
168.431
164.733
157.791
150.083
142.758
138.685
136.343
135.255
129.655
128.074
124.241
120.284
81.556
39.349
4-CMI
180.0 170.0 160.0 150.0 140.0 130.0 120.0 110.0 100.0 90.0 80.0 70.0 60.0 50.0 40.0 30.0 20.0 10.0

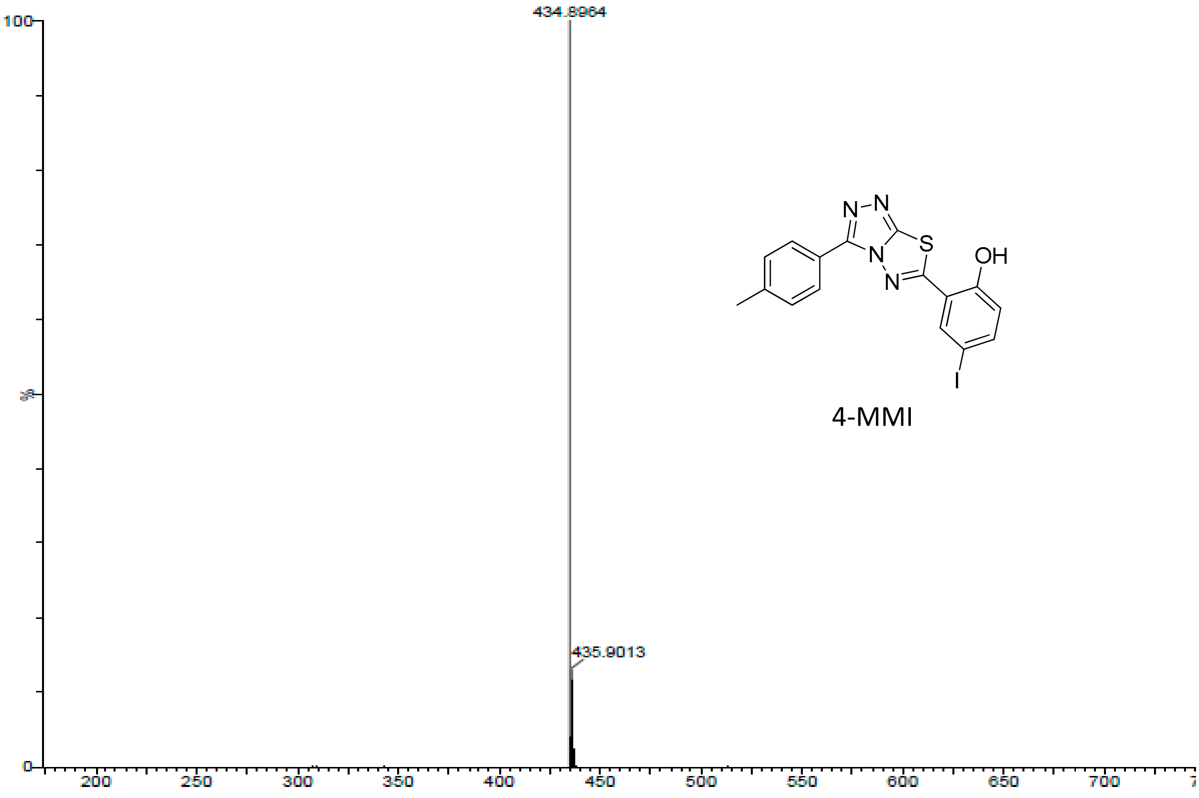
434.8964
435.9013
4-MMI
100
%
0
200 250 300 350 400 450 500 550 600 650 700

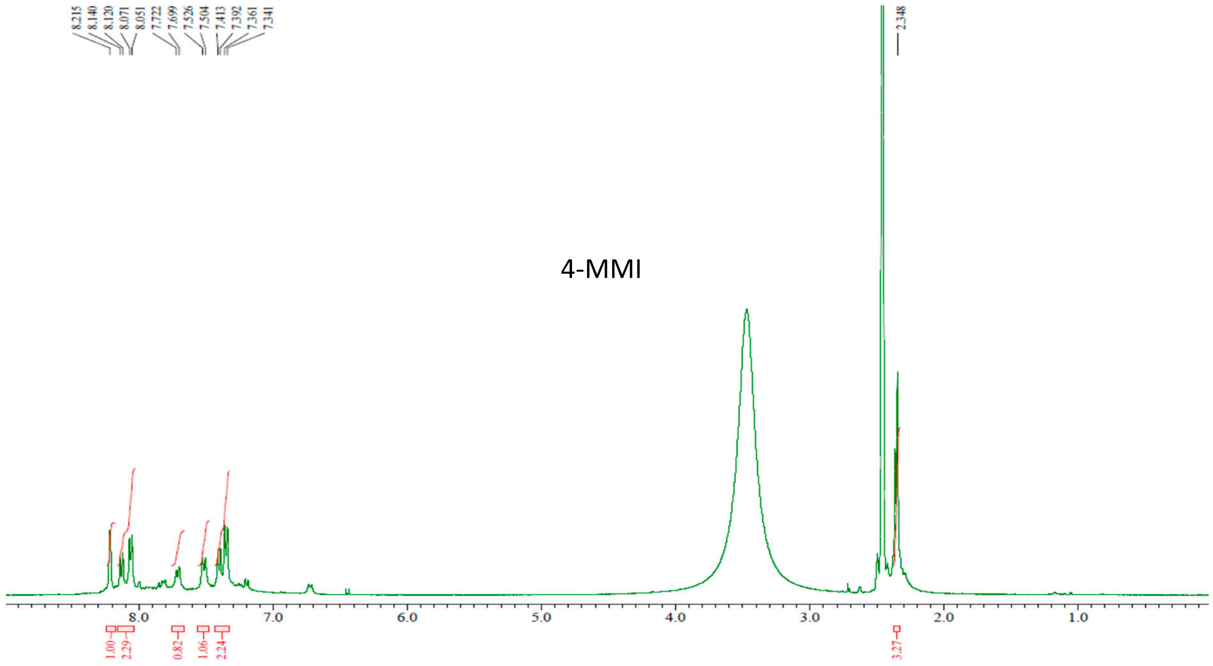

8.215
8.140
8.120
8.071
8.051
7.722
7.699
7.526
7.504
7.413
7.392
7.361
7.341
2.348
4-MMI
8.0
7.0
6.0
5.0
4.0
3.0
2.0
1.0
1.00
2.29
0.82
1.06
2.24
3.27

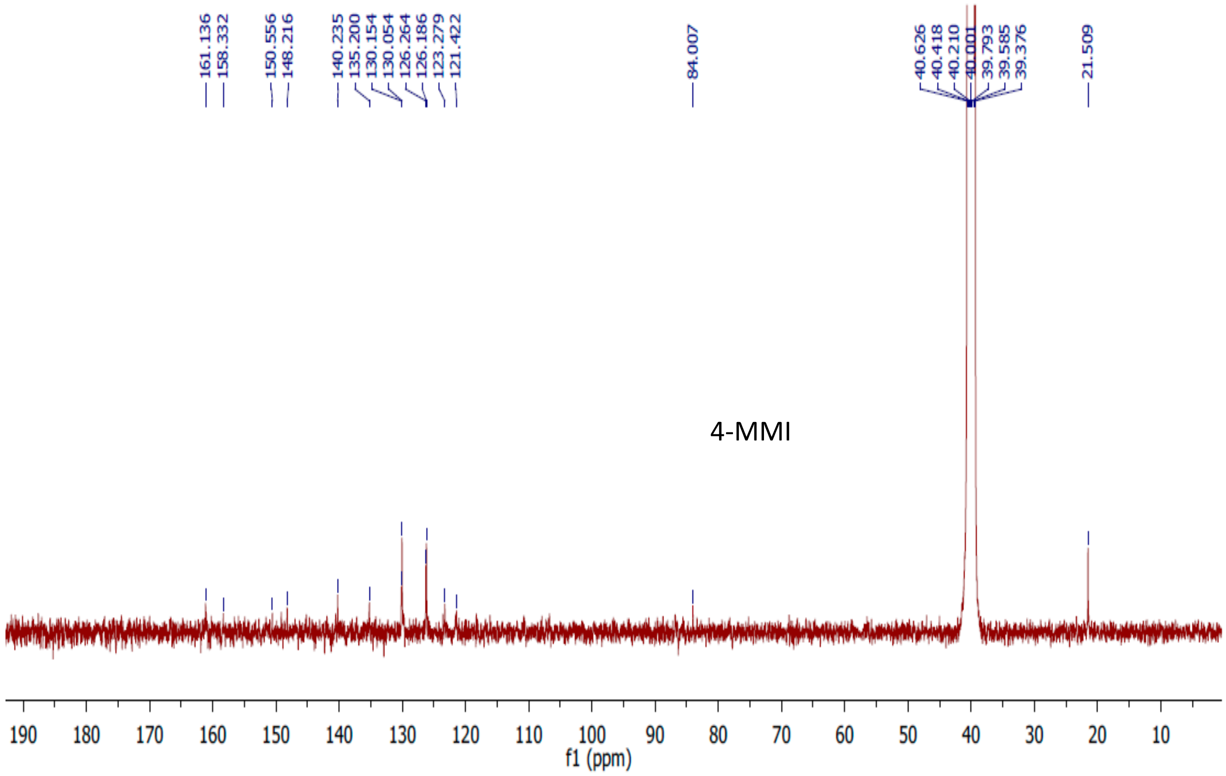

161.136
158.332
150.556
148.216
140.235
135.200
130.154
130.054
126.264
126.186
123.279
121.422
84.007
40.626
40.418
40.210
40.001
39.793
39.585
39.376
21.509
4-MMI
190
180
170
160
150
140
130
120
110
100
90
80
70
60
50
40
30
20
10
f1 (ppm)
